# Transcriptome Analysis in Prenatal IGF1-Deficient Mice Identifies Molecular Pathways and Target Genes Involved in Distal Lung Differentiation

**DOI:** 10.1371/journal.pone.0083028

**Published:** 2013-12-31

**Authors:** Rosete Sofía Pais, Nuria Moreno-Barriuso, Isabel Hernández-Porras, Icíar Paula López, Javier De Las Rivas, José García Pichel

**Affiliations:** 1 Centro de Investigación Biomédica de la Rioja, Fundación Rioja Salud, Logroño, Spain; 2 Instituto de Biología Molecular y Celular del Cáncer - Centro de Investigación del Cáncer, Consejo Superior de Investigaciones Científicas – University of Salamanca, Salamanca, Spain; University of Giessen Lung Center, Germany

## Abstract

**Background:**

Insulin-like Growth Factor 1 (IGF1) is a multifunctional regulator of somatic growth and development throughout evolution. IGF1 signaling through IGF type 1 receptor (IGF1R) controls cell proliferation, survival and differentiation in multiple cell types. IGF1 deficiency in mice disrupts lung morphogenesis, causing altered prenatal pulmonary alveologenesis. Nevertheless, little is known about the cellular and molecular basis of IGF1 activity during lung development.

**Methods/Principal Findings:**

Prenatal *Igf1^−/−^* mutant mice with a C57Bl/6J genetic background displayed severe disproportional lung hypoplasia, leading to lethal neonatal respiratory distress. Immuno-histological analysis of their lungs showed a thickened mesenchyme, alterations in extracellular matrix deposition, thinner smooth muscles and dilated blood vessels, which indicated immature and delayed distal pulmonary organogenesis. Transcriptomic analysis of *Igf1^−/−^* E18.5 lungs using RNA microarrays identified deregulated genes related to vascularization, morphogenesis and cellular growth, and to MAP-kinase, Wnt and cell-adhesion pathways. Up-regulation of immunity-related genes was verified by an increase in inflammatory markers. Increased expression of Nfib and reduced expression of Klf2, Egr1 and Ctgf regulatory proteins as well as activation of ERK2 MAP-kinase were corroborated by Western blot. Among IGF-system genes only IGFBP2 revealed a reduction in mRNA expression in mutant lungs. Immuno-staining patterns for IGF1R and IGF2, similar in both genotypes, correlated to alterations found in specific cell compartments of *Igf1^−/−^* lungs. IGF1 addition to *Igf1^−/−^* embryonic lungs cultured *ex vivo* increased airway septa remodeling and distal epithelium maturation, processes accompanied by up-regulation of Nfib and Klf2 transcription factors and Cyr61 matricellular protein.

**Conclusions/Significance:**

We demonstrated the functional tissue specific implication of IGF1 on fetal lung development in mice. Results revealed novel target genes and gene networks mediators of IGF1 action on pulmonary cellular proliferation, differentiation, adhesion and immunity, and on vascular and distal epithelium maturation during prenatal lung development.

## Introduction

Insulin-like Growth Factor 1 (IGF1) is a member of the insulin family involved in the control of tissue development and homeostasis by regulating multiple cell functions including proliferation, differentiation, survival, adhesion and migration. IGF1 acts primarily through its high affinity tyrosine kinase receptor IGF1R. IGF1 and IGF1R, in combination with the related ligand IGF2, six binding proteins with high affinity for IGFs (IGFBP 1–6), modulators of IGFs activity, and a second non-signaling receptor that reduces IGF2 signaling (IGF2R), constitute the IGF signaling system. Expression of IGF system genes is tightly regulated in a cell-type specific and spatiotemporal manner, and in addition to their endocrine actions, IGF1 and IGF2 are also frequently produced in autocrine and paracrine manners. Binding of IGFs to IGF1R causes activation of various signaling pathways, including mitogen-activated protein kinases (MAPK), PI3 kinase/Akt and STATs, which regulate their multiple functions [1]–[3].

Experimental evidence demonstrates that IGFs play key roles in prenatal lung growth and organogenesis. In humans, several mutations in both *IGF1* and *IGF1R* genes have been associated with intrauterine growth retardation [4], [5]. One of these patients, with a deletion that included the *IGF1R* gene, was reported to have lung hypoplasia [6]. IGF1 expression is deregulated in stillborn infants with respiratory and bronchopulmonary distress syndromes, and with congenital diaphragmatic hernia, diseases characterized by a severe degree of pulmonary hypoplasia and immaturity [7]–[9]. Finally, blocking IGF1R signaling in cultured human fetal lungs interfered with normal vascularization [10]. Parallel studies on genetically modified mice are contributing to better understanding the role of IGFs in pulmonary development and genetically support the fact that IGFs signaling contribute to the control of prenatal mouse lung growth and differentiation. Thus, mice carrying a homozygous null mutation of the *Igf1* gene (*Igf1^−/−^*) are born 60% of the size of their littermates and show atelectatic lungs that cause high postnatal mortality [11]–[13]. Accordingly, *Igf1r^−/−^* mice are born 45% of the size of their littermates and all of them die at birth due to immature collapsed lungs and respiratory failure [11], [13], [14]. However, perinatal *Igf2^−/−^* mice despite showing a growth deficiency similar to *Igf1^−/−^*, all survive at birth and only those born from *Igf2^−/−^* dams display a mild lung phenotype with slightly affected alveolar sacs [13], [15].

In mice, lung organogenesis prior to embryonic (E) day 16, during the pseudoglandular stage, mainly consists of branching morphogenesis with active proliferation of all cellular components. During the canalicular stage (E16.5–E17.5) there is active organization of the lung vascular bed, with numerous capillaries scattered throughout the abundant mesenchyme, and the airway epithelium cells change to columnar in proximal bronchioles and to cuboidal in the incipient airway saccules alveolar sacs. After E17.5, when the lung enters the saccular stage, cell proliferation declines and differentiation predominates. Overall, the sacculation process increases the efficiency of fluid absorption and prepares the gas exchange mechanism required at birth, which occurs in mice around E19, by means of significant morphogenetic changes and massive cell differentiation. During this period, a high proportion of distal lung epithelial cells flatten, thin out and spread to form postmitotic type I alveolar cells, while a minority remain cuboidal, acquire surfactants, and differentiate into type II cells. The distal saccular septa become thinner due to a reduction in mesenchymal cells, and the loose network of capillaries coalesces with type I cells by fusion of their respective basement membranes [16]–[19]. Despite advances made in understanding prenatal mammalian lung morphogenesis, the molecular mechanisms underlying this process remain poorly understood. IGF1 and IGF1R are broadly expressed during rodent lung organogenesis, with high levels of IGF1 preferentially present in mesenchymal cells, and IGF1R mainly found in epithelial and endothelial cells [20]–[22]. We have previously shown that IGF1 deficiency in the neonatal mouse encompasses collapsed air spaces and altered distal lung septa remodeling, characterized by changes in the expression of markers for epithelial type I and type II, and endothelial pulmonary cells [23], [24]. In addition, IGF1 was found to induce epithelium and vascular maturation in late stages of mouse fetal distal lung development [25]. Nevertheless, the cellular and molecular mechanisms by which IGF1 governs this process remain to be completely elucidated.

In the present study, we examined the lungs of *Igf1^−/−^* E18.5 mouse embryos with an inbred genetic background to analyze their prenatal lung phenotype at the cellular and molecular levels. These lungs showed disproportional hypoplasia and retarded development, characterized by a disorganized extracellular matrix and dilated capillaries. A transcriptomic analysis using microarrays identified differentially expressed genes in the *Igf1^−/−^* lungs involved in diverse cellular functions and molecular pathways reflecting immature and inflamed phenotypes. Functional validation of some of these genes using embryonic lung lobes cultured *ex vivo* revealed that the transcription factors Nfib and Klf2 and the CCN matricellular proteins Cyr61 and Ctgf mediate IGF1 actions on pulmonary maturation. Thus, the mouse mutant model, in addition and in combination with the approach using cultures of explanted embryonic lungs, may prove valuable in unveiling cellular and molecular mechanisms implicated in prenatal lung maturation and elucidating the role of IGF1 in this process.

## Results

### Postnatal Mortality and Disproportional Embryonic Lung Hypoplasia in *Igf1^−/−^* Mice Backcrossed to a C57Bl/6J Genetic Background

*Igf1^+/−^* mice containing the null *Igf1* gene locus [11] were backcrossed with the inbred C57Bl/6J strain, to avoid the highly variable lung phenotype observed in the previous mixed genetic background [23], [24]. In this new colony, 100% of the homozygous *Igf1^−/−^* mice died shortly after birth due to apparent respiratory failure, contrasting to the 60% neonatal mortality observed in the previous setting [24]. In addition, the E18.5 prenatal homozygous-null embryos showed a 42% reduction in body weight (1143.5±139.8 mg in *Igf1^+/+^ vs.* 665.2±82.3 mg in *Igf1^−/−^*), which was also higher than the previous 34% reduction reported for embryos of mixed background [13], [24]. Both results demonstrate the critical effect of the mouse strain genetic background on the phenotype of *Igf1* mutants.

**Figure 1:**
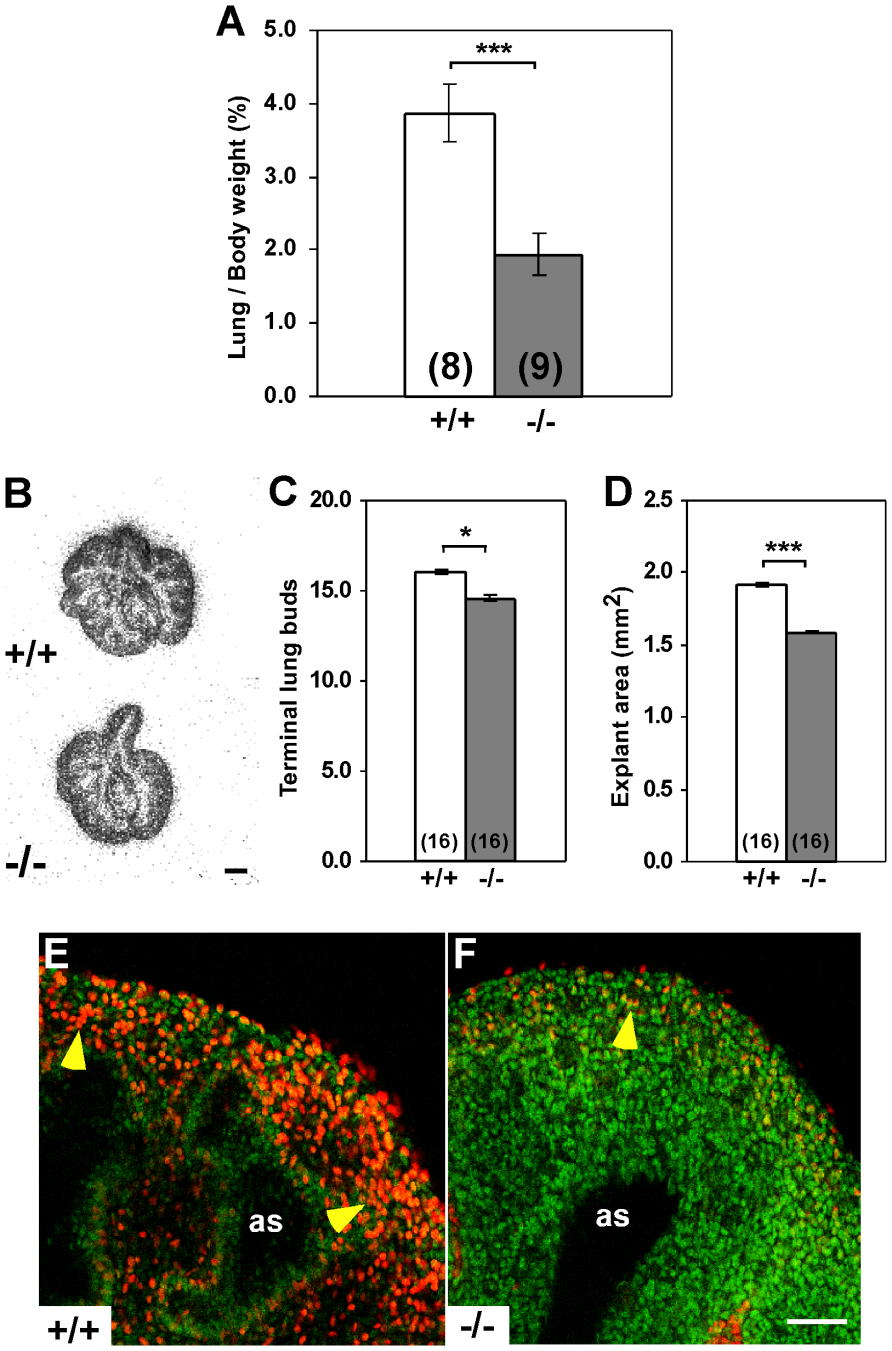
Disproportionately smaller fetal lungs in *Igf1^−/−^* mice and decreased proliferation during early pulmonary organogenesis. (**A**) Graphical representation of lung-to-body weight ratio in normal (+/+) and *Igf1^−/−^* (−/−) E18.5 fetuses (shown as mean % ± SEM). ***p<0.001 (Mann-Whitney *U* test). In parentheses, number of samples determined. Note that absence of IGF1 during embryonic development disproportionately reduces lung growth. (**B–D**) Growth deficiency in E12.5 explanted lungs after 6 h of *ex vivo* culture. Microphotographs of lung explants in (B) show a reduced number of terminal lung buds, represented in (C) (mean number ± SEM), and smaller surface, represented in (D) (mean surface ± SEM), of the *Igf1^−/−^* explants. *p<0.05; ***p<0.001 (Mann-Whitney *U* test). In parentheses, number of samples determined. (**E–F**) Cell proliferation in E12.5 explanted lungs cultured in defined medium for 48 h and pulse-chased with BrdU. Representative confocal images of whole-mount lungs analyzed from independent experiments immuno-labeled for BrdU in red (yellow arrowheads) and counterstained in green with Sytox, show more BrdU-labeled cells in *Igf1^+/+^* (E), than do *Igf1^−/−^* explanted lungs (F) (n = 5 per genotype). as, airway space. Scale bar: 250 µm in B and 30 µm in E–F.

In *Igf1^−/−^* E18.5 embryos, the lung-to-body-weight ratio was highly reduced when compared to *Igf1* ^+/+^ controls, with a significant reduction, 50% compared to their normal littermates (Fig. 1A), demonstrating that although IGF1 functions as a general growth factor for the entire organism during the embryonic period [12], [13], its role is even more crucial for proper prenatal lung growth. In an attempt to explain the retarded growth of prenatal *Igf1^−/−^* lungs, we analyzed their cell death and proliferation rates. To compare cell death levels between genotypes, lung sections were stained with DAPI to detect nuclear integrity, but no differences were found. When we tested apoptotic levels using TUNEL (TdT-mediated dUTP nick labeling) or cleaved caspase-3 immuno-staining, neither technique revealed significant differences between genotypes (data not shown). Interestingly, despite the decline in lung size, proliferation rates in E18.5 *Igf1^−/−^* lungs were higher, as shown by proliferation cell nuclear antigen (PCNA) (*Fig. S1A–B*) and BrdU immuno-staining (data not shown). These results in apoptosis and proliferation match the previously reported in embryonic lungs of the outbred *Igf1* mutants [23]. Afterwards, we analyzed the size and proliferation rates of lungs during the early stages of lung development using an organ culture approach. After 6 h of *ex vivo* culture, the flattened E12.5 *Igf1^−/−^* lung primordia showed a significant reduction in terminal lung buds and surface as compared to normal explants (Fig. 1B–D), as well as lower incorporation rates of bromodeoxyuridine (BrdU) (Fig. 1E–F). The smaller size and reduced proliferation rates found in E12.5 *Igf1^−/−^* explanted lungs suggests that the disproportional growth retardation of prenatal E18.5 *Igf1^−/−^* lungs could be a consequence of their diminished growth rates during early stages of organogenesis.

### Delayed distal differentiation and abnormalities in extracellular matrix, smooth muscle and blood vessels in the lung of *Igf1^−/−^* mice

**Figure 2:**
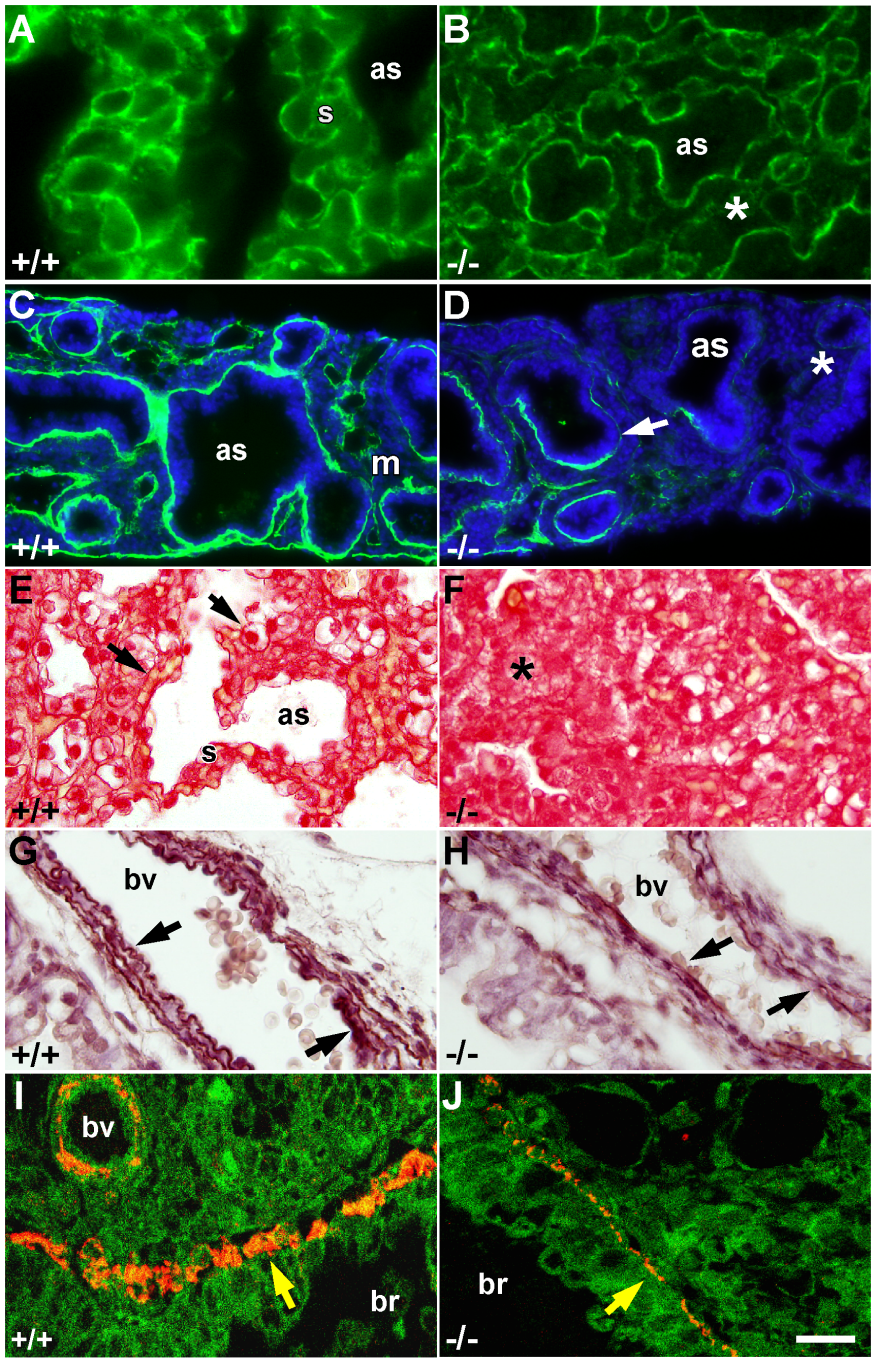
Altered extracellular matrix deposition and reduced smooth muscle actin expression in IGF1-deficient embryonic lungs. (**A–D**) Immuno-fluorescence analysis for laminin expression (green) in E18.5 embryos (A–B) (n = 4 per genotype), and E12.5 embryonic lungs cultured 96 h *ex vivo* counterstained with DAPI (blue) (C–D) (n = 3 per genotype). Cross sections of *Igf1^+/+^* lungs (+/+) show strong staining, with a regular and continuous distribution of laminin in basal membranes (A, C). In contrast, the *Igf1^−/−^* lungs (−/−) show a weaker (asterisks) or discontinuous (arrow) staining in basal membranes (B, D). (**E–F**) Collagen staining with Sirius red of E18.5 lungs. In controls (E) collagen deposition shows a well-defined and continuous fibrous reticular distribution in distal septa (arrows), while in the *Igf1^−/−^* lungs the stain is diffuse (asterisk) (F) (n = 3 per genotype). (**G–H**) Staining of elastin fibers with orcein in blood vessels of E18.5 lungs is reduced in both intensity and thickness in blood vessel walls of *Igf1^−/−^* lungs (H) (n = 4), as compared to controls (G) (n = 3) (arrows). (**I–J**) Immuno-fluorescence staining for muscle actin (monoclonal antibody clone HHF35) (red) in bronchiolar cross-sections of E18.5 lungs counterstained with Sytox (green). Thickness of bronchiolar smooth muscle is reduced in *Igf1^−/−^* lungs (J) compared to controls (I) (arrows). All images are representative of samples analyzed from independent experiments (n = 3 per genotype). as, airway space; br, bronchiole; bv, blood vessel; m, mesenchyme; s, septum. Scale bar in J corresponds to 10 µm in A–B, 50 µm in C–D and G–H, and 20 µm in E–F and I–J.

**Figure 3:**
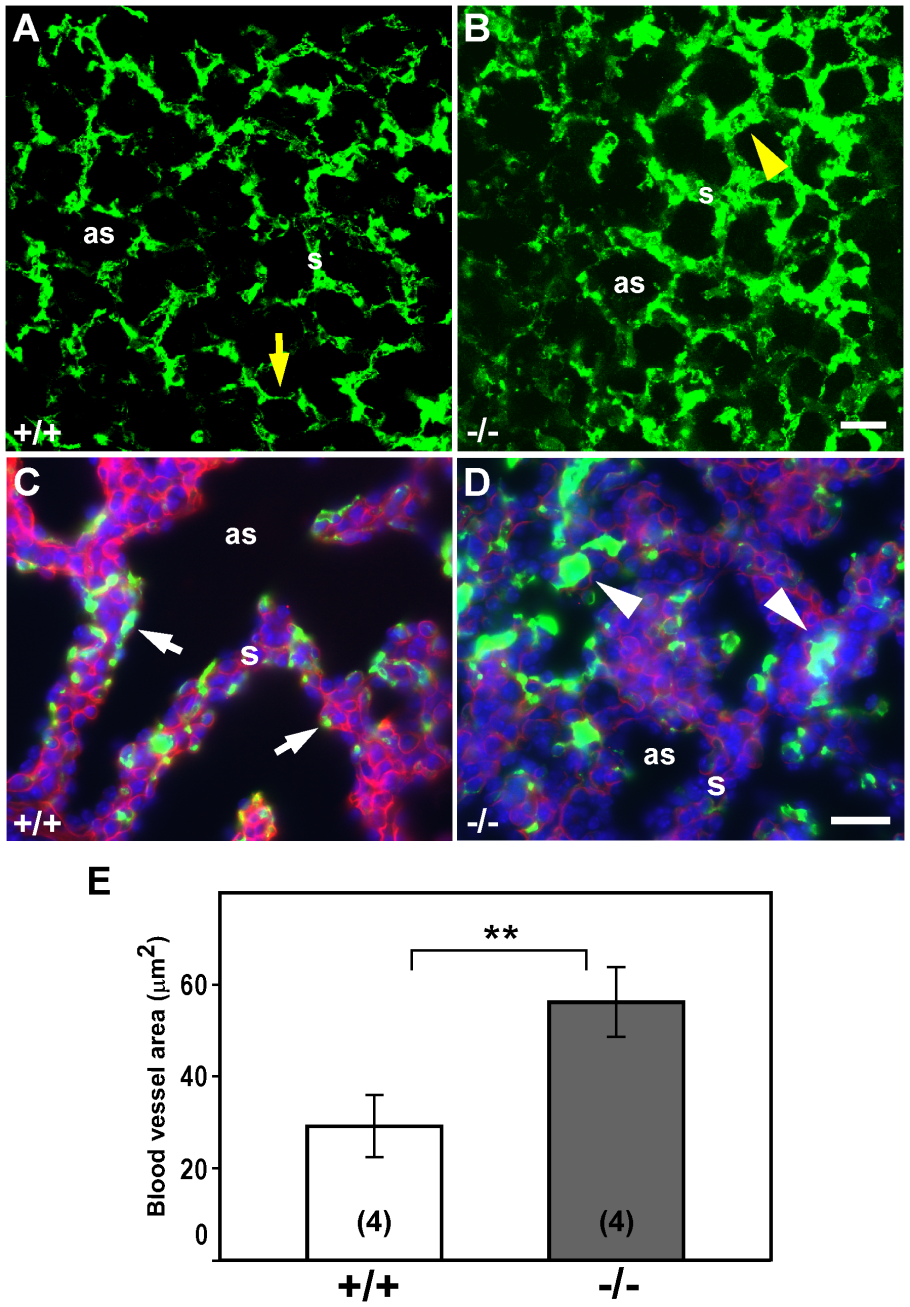
Altered lung microvasculature in E18.5 *Igf1^−/−^* embryos. Cross-sectional microphotographs of E18.5 lungs perfused with FITC-Dextran (green) to visualize the capillary network. (**A–B**) Representative three-dimensional projections of 14 consecutive 1 µm-thick confocal sections taken from whole-mounted lungs analyzed from independent experiments. Whereas in wild-type embryos (A) the fine microvasculature is normally distributed in the thin saccular septa (yellow arrow), in the *Igf1^−/−^* animals more vessels stain the thicker septa (yellow arrowhead) (B). (**C–D**) Immuno-fluorescence counter-staining with anti-laminin antibodies (red) on distal sections of FITC-Dextran perfused lungs. In *Igf1^+/+^* embryos, most of the blood vessels in the saccular septa have a thin diameter and they are located next to the saccular space (arrows in C). In the *Igf1^−/−^* lungs, the blood vessels show an increased diameter with a high proportion of them immersed in the abundant mesenchyme (arrowheads in D). Note the fainter staining of laminin in *Igf1^−/−^* lungs. (**E**) Graphic representation of the quantization of blood vessel area in both genotypes, shown as means ± SD. **p<0.01 (Mann-Whitney *U*-test). In parentheses, number of lungs evaluated. as, saccular space; s, septum. Scale bars: 25 µm in A–B and 20 µm in C–D.

Detailed histological analysis of E18.5 distal lung areas showed a dense glandular appearance with reduced air spaces, poorly defined saccular septa, presence of hyaline membrane-like content filling the air spaces, and capillaries immersed in the septal mesenchyme of *Igf1^−/−^* mice (*Fig. S1C–D*), which corresponds to the results previously reported in outbred mutant mice [11], [23]. Thus, the histology of *Igf1^−/−^* lungs better resembled an earlier pseudoglandular staging rather than the expected saccular morphology that is shown by the controls. Due to the loose pulmonary histological appearance and considering the key roles played by extracellular matrix (ECM) components during lung development as well as the possible roles of IGF1 in pulmonary ECM synthesis [26], we decided to evaluate whether ECM components were altered in the embryonic *Igf1^−/−^* lungs. Immuno-fluorescence analysis of E18.5 lungs and *ex vivo* cultured E12.5 lung primordia revealed discontinuous and weaker laminin staining in the *Igf1^−/−^* lungs (Fig. 2A–D). Although no differences were found after quantification of total lung collagen contents between genotypes (data not shown), the lung collagen-staining pattern in terminal sacs septa was found to be more diffuse and disorganized in the *Igf1*-null lungs (Fig. 2E–F). Since elastin deposition was reported to be essential for alveolar septa formation and normal alveologenesis [27], we next performed elastin staining in lung sections. Elastic fiber staining in mesenchymal areas did not reveal differences between genotypes (data not shown), but the arterial walls showed a less intense staining and a looser arrangement in thinner layers of elastin in *Igf1^−/−^* E18.5 lungs (Fig. 2G–H). Additionally, we noticed a reduction in the perivascular and parabronchial smooth muscle mass in *Igf1*-null lungs, as was demonstrated with immuno-staining of actin (Fig. 2I–J). This was not an unexpected result because IGF1 has been broadly described to induce smooth muscle proliferation, differentiation and survival [28]. To visualize lung vascular abnormalities, an FITC-labeled dextran polymer solution was inoculated into the blood stream of E18.5 embryos to visualize the lung vascular network. Angiograms, obtained by projections of confocal images captured from whole mounted lungs, denoted a disorganized distribution and thicker blood vessels in the distal alveolar septa of *Igf1^−/−^* lungs (Fig. 3A–B). Cross-sections of these FITC-dextran perfused lungs showed that blood vessels in distal parenchymal septa of *Igf1^−/−^* fetuses had thick lumens and that they were scattered in the abundant mesenchyme, away from the saccular space, whereas in normal control lungs, vessels had thin diameters and were located at the periphery of septa, surrounding saccular cavities (Fig. 3C–D and *Fig. S1C–D*). Morphometric measurements of FITC-labeled blood vessel areas revealed that section surface means were indeed significantly increased in *Igf1^−/−^* mutant lungs (Fig. 3E). These results further support the hypothesis that the lack of IGF1 alters vascularization and capillary remodeling during saccular septum maturation. Collectively, all the immuno-histological data suggest a role for IGF1 as a regulator of different aspects of prenatal lung organogenesis in mice, including saccular septum differentiation, ECM deposition, smooth muscle production and capillary remodeling.

### Differentially-expressed genes in E18.5 *Igf1^−/−^* mouse lungs

**Figure 4:**
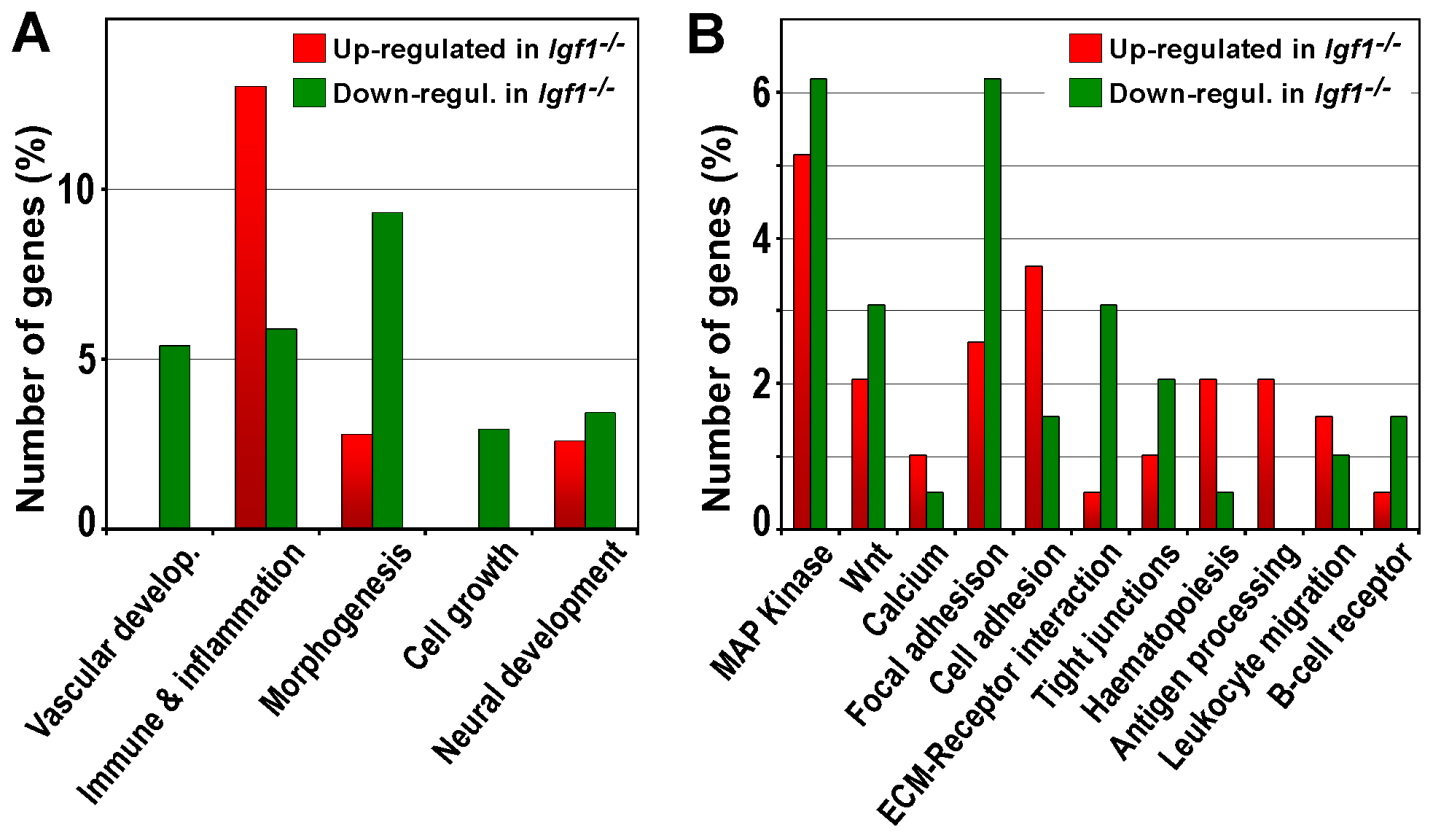
Functional annotations of IGF1-transcriptionally regulated genes obtained from RNA microarray analysis of mouse lung embryos. Analyses included differential expressed genes found in E18.5 *Igf1^−/−^* lungs with FDR<0.20. (**A**) Representation of the percentage of IGF1-regulated genes involved in biological processes classified according to either their *GO*, or (**B**) KEGG annotations. Significant biological functions (A) or molecular pathways (B) were determined using *FatiGO+* and *GeneCodis* bioinformatics applications, respectively. Bars are color-coded in red for up-regulated genes and in green for down-regulated genes. Genes included in each category of (A) and (B) are listed in *Tables S2* and *S3*, respectively.

In order to identify genes potentially involved in IGF1 function during lung development, we analyzed global RNA gene expression profiles of *Igf1^−/−^ vs. Igf1^+/+^* in E18.5 lungs. RNA extracted from lungs with both genotypes (three independent biological replicates per genotype, n = 3) was hybridized with commercial high-density oligonucleotide microarrays. After bioinformatics analysis, statistically significant changes in gene expression occurring in lungs of both genotypes were found (data submitted to *Gene Expression Omnibus*, accession number GSE17157), and two groups of gene probe-sets were defined by setting two different levels of false discovery rate (FDR) stringency. Establishing an FDR<0.20 we identified 566 genes, given by 640 probe-sets, with differential expression in *Igf1^−/−^* lungs with respect to controls. In this extended list, 200 genes were found to be up-regulated (35%) and 366 were down-regulated (65%), with a fold change in expression levels greater than 2.2 (*Fig. S2A* and *Table S1*). These results indicate that IGF1 acts as a regulator of lung gene expression. Functional annotations of these 566 genes from different databases were used to perform high-throughput bioinformatics analyses using software tools. Five significant biological functions based on *GO* (*Gene Ontology*) annotations were found using the *FatyGO+* application (Fig. 4A). Genes with vascular development, organ morphogenesis and cell growth annotations were mostly down-regulated. Strikingly, the group of genes with the highest gene representation corresponded to immune, defense and inflammatory response functions, most of them up-regulated. An additional group of genes with neural development annotations changed their expression to up or down in similar proportions. Genes included in each of these categories are listed in *Table S2*. An analysis with the *GeneCodis* application, based on *KEGG* (*Kyoto Encyclopedia of Genes and Genomics*) annotations, revealed eleven relevant molecular pathways in which IGF1-dependent genes could be participating (Fig. 4B). MAPK pathway-related genes included more than 10% of the genes assigned to significantly affected pathways. Less represented were the Wnt pathway- and calcium pathway-related genes. More than 20% of these genes were included in pathways involved in cell-cell or cell-ECM adhesion; namely, focal adhesion, cell adhesion, ECM-receptor interactions, and tight junctions. Finally, genes related to antigen processing and presentation, leukocyte trans-endothelial migration and the B-cell receptor, again pathways related to immune response and inflammation functions, were also found to be significantly represented, mostly up-regulated. The genes belonging to each of these conditions are listed in *Table S3*. We further analyzed genes with an FDR<0.20 using the Ingenuity Pathways program, obtaining a unique, fused network of 68 genes organized according to their sub-cellular localization, whose main nodes correspond to genes with previously described regulatory functions (*Fig. S3*). As expected, IGF1 appeared as a node in the extracellular space that is repressed. Additional nodes include: *VEGF, Ctgf and Mmp2* in the extracellular space, *Fn1* in the plasma membrane, *Hspa8* in the cytoplasm, and *Jun*, *JunD*, *Fos*, *Fosb*, *Egr1* and *Nr4a* transcription factors associated with nuclear functions.

When using a more stringent FDR cut-off (FDR<0.10), 59 genes (62 probe-sets) were identified as highly relevant IGF1 target genes during late lung development, all of them with a fold-change higher than 2.5 in mutant lungs as compared to controls (*Fig. S2B* and *Table S1*). A list of these genes, first organized with respect to their degree of over-expression and repression, and then subdivided into functional categories, according to *GO* annotations and published reports, is shown in *Table S4*. Of these 59 genes, 19 were up-regulated and 40 were down-regulated in *Igf1* ^−/−^ lungs. Three of the 59 genes (*Cyr61*, *Jun/AP1* and Klf6) were represented by more than one independent probe-set detected as significant in the differential expression analyses (marked with an asterisk in *Table S4*). Interestingly, some of the loci were reported to be specifically involved in lung organogenesis (*Nfib*, up-regulated; and *Klf2*, *Fgf18* and *Aqp5*, down-regulated) [29]–[32], whereas others played a more general function in tissue development (*Wnt7a* and *Klf6*, repressed) [17], [33], were related to vasculogenesis (*Cyr61*, *Ctgf*, *Vegfa*, and *Xlkd1*, repressed) [34]–[37] or to cell adhesion and ECM deposition (*Plat*, *Dpt*, *Chi3l1*, *Itgb6* and *Msln*, repressed) [38], [39]. Changes in mRNA expression of these groups of genes in *Igf1^−/−^* lungs further support involvement of IGF1 in controlling pulmonary organogenesis. Other groups of differentially expressed genes with an FDR<0.10 were linked to different specific cellular functions and/or sub-cellular compartments such as protein biosynthesis and ribosome components (*Gas5*, *Rpl30*, *Rps9*, *Rps10* and *Rpl12*, up-regulated) [40], [41], integral to the ER membrane (*Pcsk6*, and *Upk3a*, up-regulated) [42], mitochondrial enzymes (*Cox7c* and *Bdh*, up-regulated) [43], calcium metabolism (*S100a14* and *Dscr*, repressed) [44], anti-proliferative and tumor suppressors (*Scgb3a1 and Btg2*, repressed) [45], [46], chaperoning, stress response and MAPK signaling (*Hspa8*, *Nsep1* and *Map2k7*, up-regulated; *Fos*, *Nr4a1*, *Jun*, *Dusp1* and *Egr1*, down-regulated) [47]–[52], and other metabolic enzymes (*Pnpo*, *Fech* and *Fthfd*, repressed) [53]. Changes in RNA levels of these genes in *Igf1^−/−^* fetal lungs clearly indicate that IGF1 is a canonical growth factor for proper lung size development. Again, several genes known to be involved in immune/defense responses (*H2-Aa*, *Gzma*, and *Slfn1*, up-regulated; *Lcn2*, *Kitl* and *Zfp36*, down-regulated) [54]–[57] drew our attention, supporting the notion that a lack of IGF1 in the lung may result in increased levels of immunological response genes. Finally, eleven differentially expressed genes catalogued with other/unknown functions, were found repressed in *Igf1^−/−^* lungs.

We validated the gene signature obtained using microarrays by alternative experimental approaches in a different collection of samples. First, we performed a quantitative reverse transcriptase-polymerase chain reaction (qRT-PCR) on a subset of the 59-gene signature (set with FDR<0.10), consisting of 27 using RNAs from a different collection of samples (n = 4 per genotype). The qRT-PCR signals of those 27 loci, relative to the signal of ß*2-microglobin/Arbp* used as an internal control, were included in *Table S4*. All the changes obtained by qRT-PCR matched the results observed by the microarrays in terms of “up-regulation” or “down-regulation” of the corresponding genes, although some discrepancies were noted in the quantitative extent of the gene expression alterations. In all cases differences in gene expression levels between genotypes were statistically significant, as specified in *Table S4*. Expression of three additional repressed genes with FDR between 0.10 and 0.20, namely *Fn*, *Icam1* and *Atf3*, was also verified by qRT-PCR (*Table S5*). These genes were chosen based on their previously reported role in lung development: Fn1 has been implicated in lung branching morphogenesis [58], Icam1 expression was described in endothelial and alveolar type I epithelial cells [32], and the immediate-early response transcription factor, Atf3, implicated in lung injury recover [59], also mediates p38 MAPK signaling, an enzyme extensively involved in lung morphogenesis and differentiation [60]–[62].

**Figure 5:**
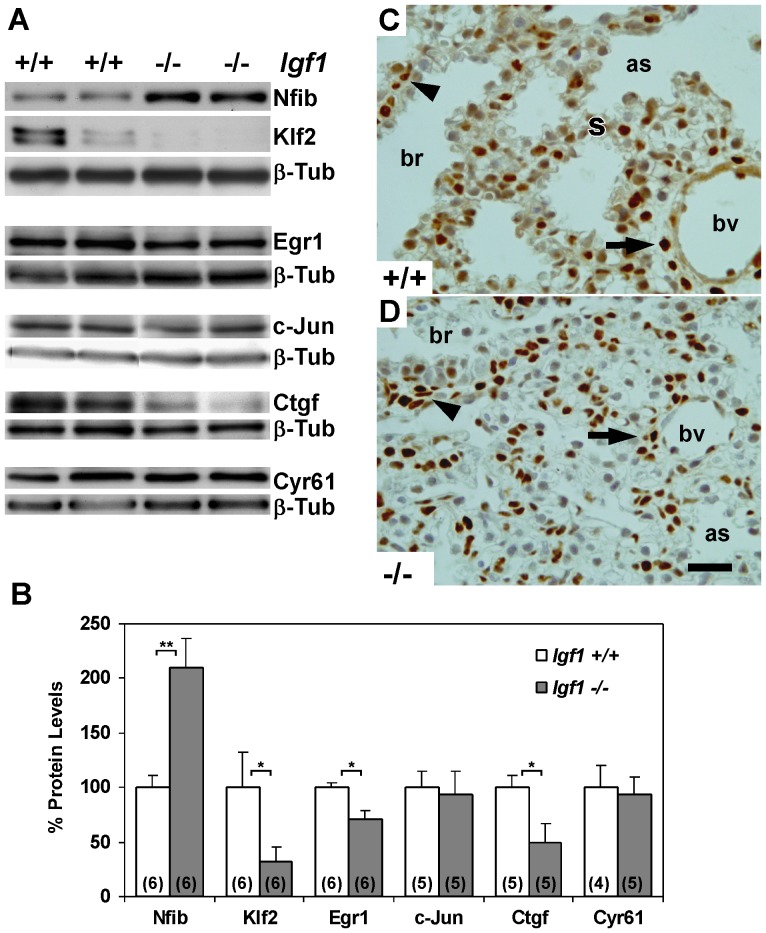
Protein levels of selected regulatory genes found with differential expression in microarrays of RNA. (**A**) Representative Western blots for Nfib, Klf2, Egr1 and c-Jun transcription factors as well as Ctgf and Cyr61 matricellular growth factors in E18.5 *Igf1^+/+^* and *Igf1^−/−^* lungs. β-Tubulin was used as a loading control. (**B**) Graphical representation of densitometric measurements of specific band signals after total protein loading normalization with ß-Tubulin. *Igf1^+/+^* relative values were taken as 100%. In parentheses, number of samples determined. Increased levels of Nfib in *Igf1^−/−^* lungs were statistically significant (**p<0.01), and decreased levels of Klf2, Egr1 and Ctgf were also found to be significant (*p<0.05) (Mann-Whitney *U*-test). (**C–D**) Representative immuno-staining for Nfib in lung cross-sections analyzed from independent experiments. Note high levels of Nfib nuclear expression in subsets of mesenchymal cells surrounding blood vessels (arrows) and bronchioles (arrowheads), more evident and with a flattened morphology in *Igf1^−/−^* (n = 4) than in *Igf1^+/+^* (n = 3) lungs. as, saccular space; br, bronchiole; bv, blood vessel; ß-Tub, ß-Tubulin; s, septum. Scale bar: 20 µm.

mRNA microarray data of *Igf1^−/−^* lungs revealed changes in gene expression levels which were validated at the protein level using immunoblotting of whole lung extracts to test for certain regulatory proteins, especially transcription and growth factors. We chose proteins that were either previously implicated in lung organogenesis or identified with functions regulated by IGF1, such as the transcription factors Nfib, Klf2, Egr1 and c-jun, and the matricellular proteins Cyr61/CCN1/Igfbp10 and Ctgf/CCN2/Igfbp8 [30], [63]–[68]. We observed significantly increased protein levels of Nfib and significantly decreased levels of Klf2, Egr1 and Ctgf in the *Igf1^−/−^* lungs, all of which correlated to their changes in mRNA levels. However, c-Jun and Cyr61 protein levels did not change to match their reduced mRNA levels in the *Igf1^−/−^* lungs (Fig. 5A–B). Nfib expression was further evaluated by immuno-staining of *Igf1^−/−^* lung sections. Nuclear expression of Nfib, mostly observed in subsets of mesenchymal cells and smooth muscle cells surrounding bronchioles and arterioles of normal lungs (Fig. 5C) as previously reported [29], displayed a similar pattern in the highly abundant mesenchymal cells of *Igf1^−/−^* lungs (Fig. 5D). Altogether, these data contribute to our understanding of the molecular basis of delayed lung maturation in the context of IGF1-deficiency, where IGF1 affects the expression of key regulatory genes in lung organogenesis.

### Expression of IGF System Genes and Activation of their Signaling Mediators in the *Igf1^−/−^* Lungs

**Figure 6:**
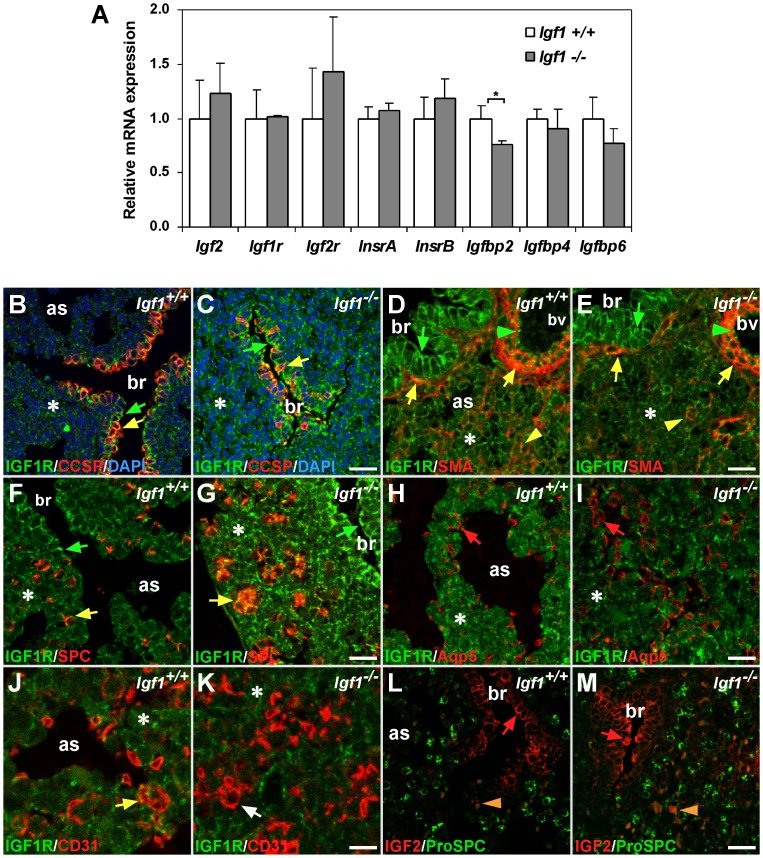
Expression of IGF system genes in the E18.5 lung. (**A**) mRNA expression levels of *Igf2, Igf1r, Igf2r, Insr* (splice variants A and B), *Igfbp2*, *Igfbp4* and *Igfbp6* analyzed by qRT-PCR in *Igf1^+/+^* and *Igf1^−/−^* lungs (n = 4 per genotype). *Gapdh* was the endogenous control gene. Only *Igfbp2* mRNA levels were significantly reduced in *Igf1^−/−^* lungs (*p<0.05) (Mann-Whitney *U*-test), however note the slightly increased *Igf2*, *Igf1r*, *Igf2r*, *InsrA* and *InsrB* and slightly reduced *Igfbp4* and *Igfbp6* mRNA mean levels in *Igf1^−/−^* lungs. (**B–K**) Immuno-staining of IGF1R (green labeling) counterstained in red with lung cell-type specific markers. IGF1R staining was high in the bronchiolar epithelium (green arrows), but also found scattered throughout the distal parenchyma (asterisks). No major differences were noted between genotypes. (B–C) Bronchiolar epithelium showed strong staining for IGF1R (green arrows), with the highest levels co-localizing in CCSP^+^ Clara cells (yellow arrows). (D–E) IGF1R stained vascular endothelial cells (green arrowheads) and co-localized with SMA (smooth muscle actin, clone 1A1 antigen) in peribronchiolar and perivascular smooth muscle (yellow arrows), and in scattered cells of lung parenchyma (yellow arrowheads). (F–G) Co-stain with Pro-SPC (SPC) showed IGF1R co-localization in many type 2 pneumocytes (yellow arrows), more randomly distributed in controls (F) and with a more acinar-like organization in *Igf1^−/−^* lungs. (H–I) Type 1 epithelial cells, stained with Aqp5, did not co-stain with IGF1R (red arrows). (J–K) CD31 endothelial marker co-localized with IGF1R in some parenchymal endothelial cells of *Igf1^+/+^* lungs (yellow arrow in J), but not in *Igf1^−/−^* (white arrow in K). (**L–M**) Immuno-staining for IGF2 expression (red labeling) was positive in bronchiolar epithelial cells of both genotypes (red arrows). EDTA antigen retrieval for IGF2 caused unspecific refringent signal on red blood cells (orange arrowheads). All confocal images in B–M are representative of samples analyzed from independent experiments. as, airway space; br, bronchiole; bv, blood vessel. Scale bars: 25 µm in B–C and F–G, 17 µm in D–E and L–M, 12 µm in H–I and 8 µm in J–K.

To determine if the lack of IGF1 alters the expression of IGF system genes at the mRNA level, we analyzed the expression of *Igf2*, *Igf1r*, *Igf2r*, *Insr* (splice variants A and B), *Igfbp2*, *Igfbp4* and *Igfbp6*, in a different set of *Igf1^+/+^* and *Igf1^−/−^* E18.5 lungs (n = 4 per genotype) using qRT-PCR. With the exception of *Igfbp2*, which showed a minor, but significant reduction (0.8-fold in *Igf1^−/−^* respect to WT), none of the other genes showed significant differences in transcript levels between genotypes (Fig. 6A). These results concur with the expression profiling results obtained using microarrays, where none of these genes were differentially expressed.

To better understand the pulmonary phenotype of *Igf1^−/−^* mice, we compared IGF1R and IGF2 expression patterns in E18.5 lungs of control and *Igf1^−/−^* lungs by immuno-staining. We chose IGF1R as the main cell autonomous mediator of IGF1 action, and IGF2 as the alternative ligand signaling through IGF1R. To identify their cellular localization, we performed immuno-co-staining with specific markers for different pulmonary cell types [17] (Fig. 6B–M). We found a ubiquitous staining for IGF1R throughout the entire control lungs, although with important differences in staining intensity among different cell compartments and cell types, as it was reported [21]. The mutant lungs showed a similar pattern, with only minor differences (Fig. 6B–K). In the proximal lung we noticed prominent staining for IGF1R in the entire bronchiolar epithelium (Fig. 6B–C, D–E and F–G). Among bronchiolar epithelial cells, the higher levels of IGF1R corresponded to Clara cells, which specifically express the Clara Cell Secreted Protein (CCSP) marker (Fig. 6B–C). Strong IGF1R staining was also found in endothelial cells of large vessels (Fig. 6D–E), co-localized with smooth muscle actin (SMA) in areas of perivascular and peri-bronchiolar smooth muscles (Fig. 6D–E). In the distal lung, IGF1R staining was found scattered throughout the entire parenchyma, co-localized with SMA-positive cells (Fig. 6D–E). IGF1R positive cells coincided with many Surfactant Protein C Precursor (ProSPC)-stained cells, a marker that identifies type 2 alveolar epithelial cells (Fig. 6F–G), but did not co-localize in cells positively stained for Aquaporin 5 (Aqp5), a type 1 pneumocyte marker (Fig. 6H–I). Interestingly, we found a frequent co-staining of IGF1R with the CD31/PECAM capillary endothelial marker in *Igf1^+/+^* lungs (Fig. 6J), a pattern that was not so evident in the *Igf1^−/−^* samples (Fig. 6K). IGF2 positive immunostaining was intense in lung bronchiolar epithelium and absent in the distal pulmonary parenchyma, with no differences between genotypes (Fig. 6L–M).

**Figure 7:**
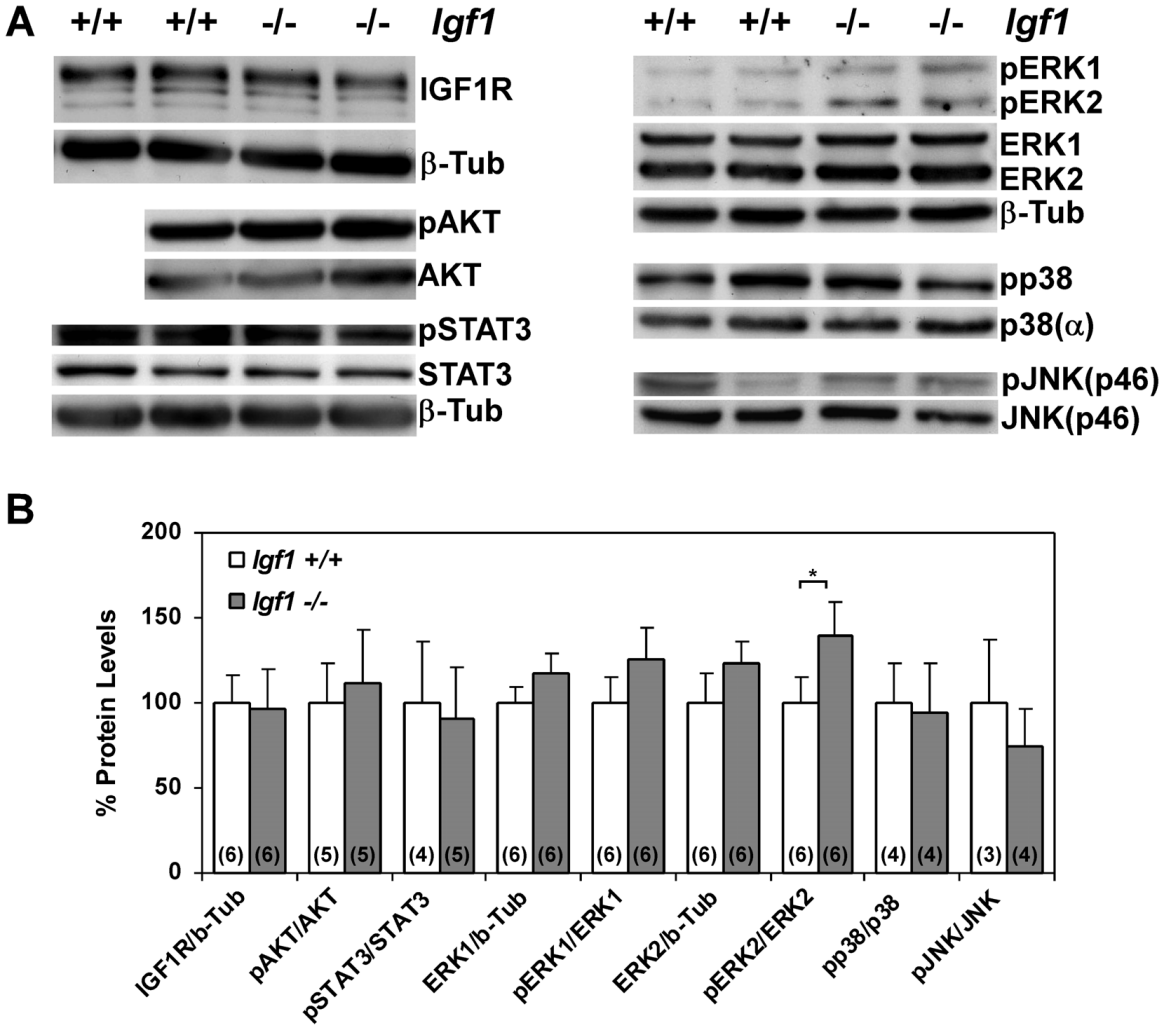
Levels of IGF signaling mediators in prenatal *Igf1^−/−^* lungs. (**A**) Representative Western blots of total protein extracts for total IGF1R, phosphor-(p)-Akt and total Akt, pSTAT3 and total STAT3, pERK1/2 and total ERK1/2, pp38 and total p38-alpha and pJNK and total JNK (p46), using two representative samples from both normal and *Igf1^−/−^* E18.5 lungs. Activation levels were determined using phosphor-specific antibodies. β-Tubulin was additionally used as a protein loading control (bottom panels). (**B**) Western blot band densitometric measurements of after total protein loading normalization, using either ß-Tubulin or total content of each protein when evaluating phosphorylation levels with phosphor-specific antibodies. *Igf1^+/+^* relative values were taken as 100%. In parentheses, number of Western blots quantified per genotype. Increased levels of pERK2 with respect to total ERK2 was found to be significant (*p<0.05) (Mann-Whitney *U*-test). ß-Tub or b-Tub, ß-Tubulin.

Additionally, we compared total protein content and activation levels by phosphorylation of different IGF1 signaling mediators by immunoblotting of proteins obtained from lungs of both genotypes. When we evaluated the expression of IGF1R, we did not find differences between genotypes (Fig. 7A–B). Although downstream molecular pathways for IGF1/IGF1R signaling include the canonical Akt mediator, and activation of STAT3 transcription factor, both described to be involved in lung cells and lung development [69], [70], we did not find differences in their expression or activation levels (Fig. 7A–B). An alternative recognized IGF1/IGF1R signaling mediator is the MAPK pathway, reported with implications in lung development [60], [71]–[73], and upon genes related to this pathway found to be differentially expressed in the microarray analysis of *Igf1^−/−^* lungs (Fig. 4B). Thus, when we analyzed total protein and phosphorylation levels of ERK1/2, p38 and JNK MAP kinases in lung extracts, we noticed an increase in total mean expression and phosphorylation levels of both forms of ERK, but only the level on pERK2 activation level was statistically significant (Fig. 7A–B). In addition, we did not find changes in levels of expression or activation in p38 or JNK (p46) MAP kinases (Fig. 7A–B). Altogether, these results suggest that IGF1 deficiency during embryonic development did not have compensatory effects on IGF system gene expression levels or their signaling pathway components, although this deficiency did lead to an increased activity of ERK2 MAP kinase.

### IGF1 deficiency increases the presence of inflammatory markers in prenatal lungs

**Figure 8:**
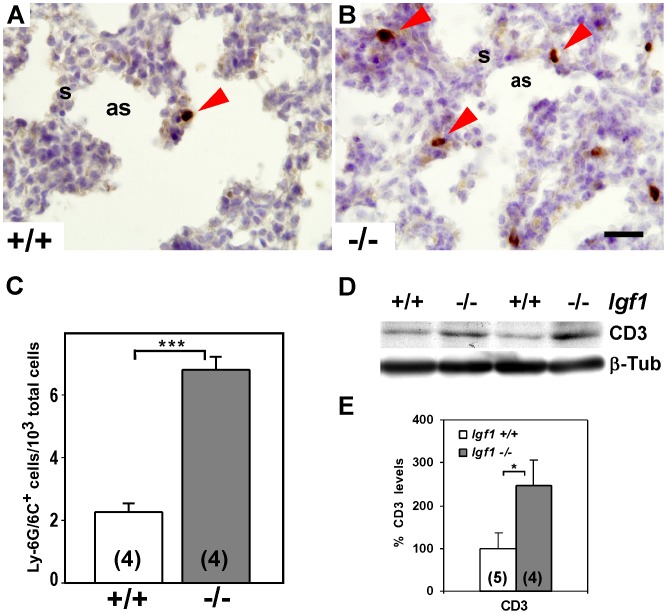
Prenatal IGF1-deficiency during lung development increases the presence of pulmonary inflammatory cells. (**A–B**) Representative immuno-staining for Ly-6G/6C (Gr1) from independent experiments in paraffin cross-sections of E18.5 lungs. *Igf1^−/−^* lungs (B) have increased numbers of cells with positive staining as compared to wild type littermates (A) (red arrowheads) (n = 4 samples/genotype). (**C**) Graph representing quantization of Gr1-positive cells in both genotypes, showing a three-fold increase in *Igf1^−/−^* lungs. Graphs represent means ± SEM. ***p<0.001 (Mann-Whitney *U*-test). (**D**) Total CD3 protein levels, a marker for T-lymphocytes, analyzed by Western blot in two samples of each genotype. (**E**) Representation of CD3 densitometric mean signal (± SEM) after total protein loading normalization with ß-Tubulin. Five (*Igf1^+/+^*) and four (*Igf1^−/−^*) different samples were used. Increased levels of CD3 were significant (*p<0.05) (Mann-Whitney *U*-test). as, saccular space; s, septum; ß-Tub, ß-Tubulin. Scale bar: 20 µm in A–B.

To investigate whether the increase in expression of immune and inflammatory genes might correspond to an accumulation of inflammatory cells in mutant lungs, we analyzed the presence of inflammatory cells in *Igf1^−/−^* lungs. Using immuno-staining for the Ly-6G/6C antigen, a cell surface marker of granulocytes and cells of myeloid lineage [74], we found a three-fold increase in the number of stained cells on the mutant lungs (Fig. 8A–C). Immunoblotting, of the T cell-specific CD3 antigen also revealed a significant increase in CD3 protein levels in the *Igf1^−/−^* lungs, pointing to an increased presence of T-cells (Fig. 8D–E). These results indicate that the lack of IGF1 during embryonic development could induce the recruitment of inflammatory cells to the lung.

### IGF1 functionally rescues saccular septum maturation in *ex vivo* cultured lungs by regulating expression of Nfib, Klf2, Cyr61 and Ctgf

**Figure 9:**
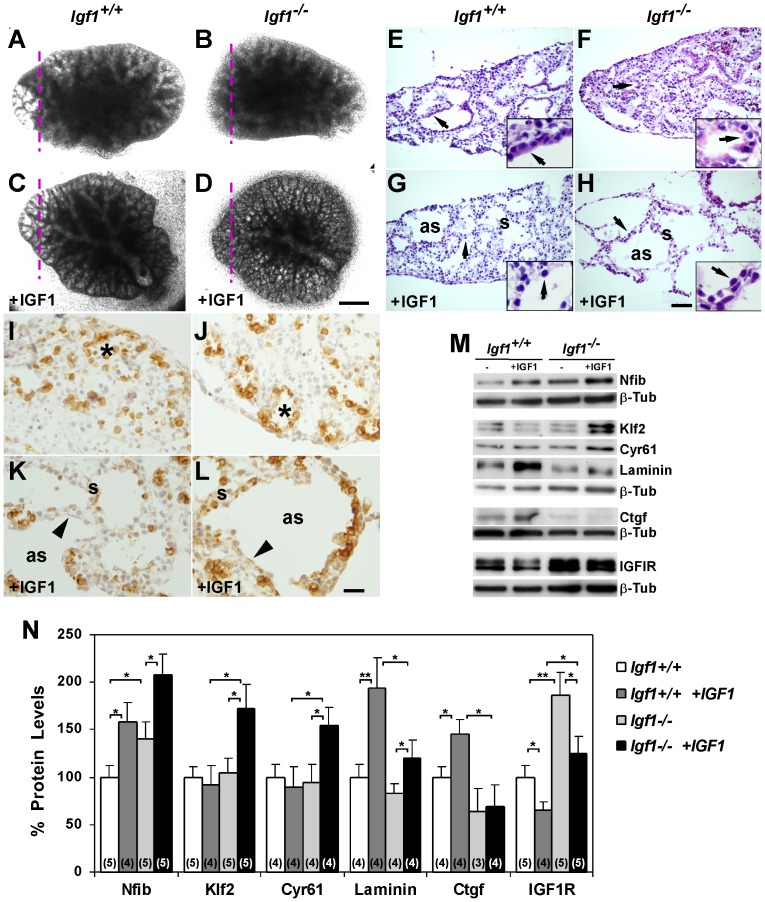
IGF1 induces alveolar morphogenesis and expression of target genes in prenatal lungs cultured *ex vivo*. E16.5 *Igf1^+/+^* and *Igf1^−/−^* lung lobes were cultured in defined medium for 96 h in the presence or absence of recombinant IGF1 (100 ng/mL). (**A–D**) Images of cultured lobes showing the effect on explant morphogenesis induced by addition of IGF1. Note the differences in morphology shown by *Igf1^−/−^* explants treated with IGF1 (D) (n = 10 per condition). Purple dashed lines indicate sectioning planes in E to L. (**E–H**) H&E staining on sections of the cultured lungs (n = 4 per condition). Untreated explants show compact tissue with undefined septa and reduced spaces mainly lined by cuboidal epithelium (arrows in E–F). IGF1 treatment opens tissue spaces, narrows septa and flattens the epithelium (arrows in G–H), with a more pronounced effect on *Igf1^−/−^* explants (H). Insets in E–H are high magnifications of lung epithelium (arrows), demonstrating that it becomes thinner and flatter in both genotypes of IGF1-treated cultures. (**I–L**) Immuno-histochemical staining for Pro-SPC on lung explants. The high proportion of cubic positive cells lining the reduced aerial spaces in non IGF1-treated explants (asterisks in I–J), decrease in proportion in the epithelium of IGF1-treated tissues (arrowheads in K–L) (n = 2 per condition). All images in A–L are representative of samples analyzed from independent experiments. (**M**) Immunoblots for IGF1 target gene expressions in explanted lungs. (**N**) Densitometric representation of gene expression levels after total protein loading normalization with ß-Tubulin (ß-Tub). In parentheses, quantified Western blots. IGF1 addition to explants increased levels of Nfib and laminin in lungs of both genotypes, Ctgf in *Igf1^+/+^*explants, and Klf2 and Cyr61 in *Igf1^−/−^* samples, but it reduced levels of IGF1R in both genotypes. (*p<0.05; **p<0.01) (Mann-Whitney *U*-test). as, air space; s, septum. Scale bar corresponds to 500 µm in A–D; 50 µm in E–H, and 7 µm in their insets; 20 µm in I–L.

The *Igf1^−/−^* lung histological and molecular alterations described above are a consequence of cumulative changes due to the absence of growth factor throughout embryogenesis, and possibly counteracted by compensatory mechanisms that could partially mask the precise role of IGF1 in prenatal pulmonary organogenesis. To validate and demonstrate the direct action of this growth factor on prenatal lung organogenesis, we studied the effects of adding exogenous IGF1 to E16.5 explanted lobes of normal and *Igf1^−/−^* lungs. These explants were cultured *ex vivo* in defined medium using an experimental setup that allows E16.5 mouse lung tissue to growth at the air–liquid interface, in a similar manner as previously described [75]–[77]. Explanted lungs of both genotypes grew in culture proportionally to their original size (Fig. 9A–B). Addition of IGF1 to the medium barely changed the morphology of wild-type cultured lungs (Fig 9C). However, in *Igf1^−/−^* lungs, IGF1 induced changes in morphology by increasing the proportion of clear areas in the distal lobes (Fig. 9D). Histological analyses of the normal explants revealed immature distal clear spaces transected by thick septa that were still lined with a high proportion of cubic epithelium (Fig. 9E and inset). As expected, *Igf1^−/−^* explants showed reduced terminal spaces, barely exhibited defined distal parenchymal septa and demonstrated a poorly differentiated epithelium (Fig. 9F and inset). Addition of IGF1 to cultured wild type-lungs led distal septa to become narrowed and their epithelium to flatten (Fig. 9G and inset). Strikingly, the effects of IGF1 were heavily increased in the *Igf1^−/−^* explanted lungs, which had thinner septa (2 to 6 cells thick), scarce mesenchyme, and a reduced presence of cubic epithelial cells (Fig. 9H and inset). Positive staining for pro-SPC in a high proportion of epithelial cells organized in acinar-like structures in the non-IGF1-treated explants revealed their more immature staging (Fig. 9I–J). Addition of IGF1 reduced the proportion of cells stained for pro-SpC, and increased the proportion of pro-SpC-negative cells with flatten morphology (Fig. 9K–L). Thus, IGF1 addition to explanted lungs induced pulmonary distal parenchymal septum maturation *in vitro*, an action more obvious in *Igf1^−/−^* explants, which likely resembled the maturation stage described *in vivo* in the normal lungs at the E18.5 developmental stage (*Fig. S1C*) [23].

Next we checked if *in vitro* IGF1 action on the explanted lungs induced changes in expression levels of the genes that we proposed above as candidate targets of IGF1 action during lung organogenesis. When we analyzed Nfib, Klf2, Ctgf and Cyr61 protein levels bound to laminin in the cultured lungs, we found that exogenous IGF1 elicited different results depending on the gene analyzed (Fig. 9M–N). When we cultured explants without IGF1 we only found significant increased levels of Nfib in the *Igf1^−/−^* explants respect to the *Igf1^+/+^*, although there were no differences for Klf2, Cyr61, laminin and Ctgf (Fig. 9M–N). It is noteworthy that this increase in Nfib reflects the results found in E18.5 native lungs (*Table S4* and Fig. 5). Addition of exogenous IGF1 to *Igf1^+/+^* explants elicited a significant increase in the expression of Nfib, laminin and Ctgf, but did not change levels in Klf2 and Cyr61. In contrast, in *Igf1^−/−^* cultured lungs all proteins, with the exception of Ctgf, had significantly increased levels after addition of IGF1 (Fig. 9M–N). When we looked for Nfib immuno-localization in the cultured lungs, we noticed that in the IGF1-untreated explants the Nfib-positive cells were randomly distributed in the tissue, preferentially in the mesenchymal compartment (*Fig. S4A–B*). Interestingly, in the IGF1-treated tissues, cells with a stronger signal for Nfib mainly aligned under the epithelium, a pattern better noticed in the *Igf1^−/−^* explants (*Fig. S4C–D*). Independently of whether exogenous IGF1 was added, IGF1R protein levels were significantly higher in *Igf1^−/−^* cultured lungs than in *Igf1^+/+^* ones. The addition of IGF1 caused a reduction in IGF1R expression in explants of both genotypes, probably due to feedback inhibition, as previously reported in other biological systems (reviewed in [78]). Taken together, these results further support the idea that IGF1 induces distal lung prenatal maturation, which acts on epithelial and mesenchymal cells by regulating gene expression of transcription factors Nfib and Klf2 and matricellular proteins Cyr61 and Ctgf.

## Discussion

We studied IGF1 function during embryonic pulmonary organogenesis by analyzing the lung phenotype of the *Igf1^−/−^* mouse in a C57Bl/6J background. For this aim we compared distinct features of *Igf1^−/−^* and *Igf1^+/+^* prenatal lungs: i) histopathology; ii) pulmonary transcriptome changes and their validation at the protein level on selected regulatory genes as possible targets of IGF1 action; iii) expression of IGF-system genes; and iv) histopathological and molecular effects of exogenous IGF1 on *ex vivo* explanted lungs. This study allowed us to identify molecular pathways and novel regulatory genes in which IGF1 is involved during mouse lung embryonic development.

In mice, prenatal lung maturation is strain dependent, as demonstrated by Xu *et al.* who compared transcriptomic expression patterns in C57Bl/6J and A/J mice [79], and it would explain why *Igf1^−/−^* mice have a highly variable pulmonary phenotype in mixed genetic backgrounds [23], [24]. The new C57Bl/6J inbred background had beneficial consequences which unified and emphasized their lung phenotype, and led us to investigate additional aspects of IGF1 function during pulmonary organogenesis. It is feasible that the uniform maturation delay shown by *Igf1^−/−^* embryonic lungs in this new genetic context contributes to increasing their mortality at birth and further corroborates that the *Igf1* mutation in mice is dependent on the genetic background and causes higher detrimental phenotypes in inbred backgrounds, as previously reported [11], [24].

Although it is now clear that IGF1 and IGF2, signaling through IGF1R, participate in the developmental control of fetal lungs, this report clearly demonstrates that the role of IGF1 in lung organogenesis is more critical than that described for IGF2. Thus, IGF2 deficiency only afforded a subtle lung differentiation delay, characterized by thicker and disorganized distal lung septa, which did not compromise neonatal survival [15]. Furthermore, *Igf2^−/−^* prenatal lungs showed increased expression of IGF1, probably in an attempt to compensate IGF2 deficiency [15]. Conversely, as shown in this report, IGF1 pulmonary function was characterized by a more severe lung differentiation impairment, which was not associated with compensatory changes in the expression of IGF2. Among the different IGF-system genes tested, we only found decreased levels of IGFBP2 mRNA in the mutant lungs. These data contrast with results found in *Igf1^−/−^* cochleae, where its expression was increased [80], and emphasized the organ specific actions of IGF1 during mouse organogenesis. It is striking that the high levels of IGF1R observed in bronchiolar epithelium do not entail major histological or molecular modifications related to this cell compartment in *Igf1^−/−^* lungs. It is possible that high levels of IGF2 revealed in the bronchiolar airway epithelium could compensate IGF1 deficiency in the mutants and therefore maintaining a normal IGF1R signaling and the absence of abnormalities. However, more extended studies will be needed to conclusively demonstrate this assumption.

Akt, STAT3 and MAP kinases, known as canonical IGF signaling mediators, were previously implicated in controlling cell proliferation, growth, survival and differentiation during lung development [60], [69]–[73]. Furthermore, prenatal mouse cochleae of *Igf1^−/−^* embryos showed decreased activation of prosurvival Akt and proliferation-associated ERK1/2 kinases, and increased levels of the stress kinase p38 [80]. However, although we found a bundle of genes related to the MAP kinase signaling pathway with differentially expressed mRNA levels, we only found a significant increase of ERK2 activation in *Igf1^−/−^* lungs. Since we had previously reported elevated levels of ERK1/2 activation in highly mitotic and undifferentiated lungs of *Lif/Igf1* double knockout mice [23], ERK2 activation seems to be a tissue specific target of IGF1-deficiency in the lung.

E18.5 *Igf1^−/−^* lungs elicited disproportional prenatal pulmonary hypoplasia, demonstrating that IGF1 plays a differential role in organ growth, especially affecting the lung. The disproportional lung size of prenatal E18.5 *Igf1^−/−^* lungs seems to be an outcome of their reduced growth rates and branching morphogenesis found during early stages of organogenesis. Thus, E12.5 *Igf1^−/−^ ex vivo* explanted lungs showed a significant reduction in size and proliferation, in parallel to a decreased number in terminal lung buds. Disproportionate effects on postnatal organ growth were previously reported in *Igf1^−/−^* mice, where IGF1 deficiency affected the growth of cochleae to a lesser extent, but had a greater effect on the lungs, whose growth was hindered more [48], [49]. The ratio of lung to body weight was also greatly reduced in *Igf1r^−/−^* prenatal embryos [14]. One possible reason for this reduced lung size could be massive cell death due to the lack of IGF1/IGF1R signaling, as was previously described in prenatal lungs of *Igf1r^−/−^* mutant mice, and postnatal cochleae of the *Igf1^−/−^* mutants [14], [81]. However, both this study and our previous report [23], failed to demonstrate changes in apoptotic rates of *Igf1^−/−^* prenatal lungs, and therefore suggesting that IGF1 would not play a major role in prenatal lung survival. Concurring with these results found in mice, there is evidence that IGF signaling also plays roles in prenatal lung growth in humans. Thus, mutations in *IGF1* and *IGF1R* genes have been found in patients with general intrauterine growth retardation [4], [5], and lung hypoplasia [6].

Increased proliferation rates revealed by PCNA and BrdU markers in E18.5 *Igf1^−/−^* lungs agree with increased RNA levels of ribosomal proteins, known to be involved in protein biosynthesis during mitosis [41], and with ERK2 kinase activation, reported as parallel proliferation in early and immature stages of lung development [23], [73], [82]. This increased prenatal proliferation could be explained as an attempt to compensate for the reduced proliferation and hypoplasia shown by *Igf1^−/−^* lungs at earlier stages, and it may contribute to reducing airway space and lung collapse, further resembling the findings in *Igf1r^−/−^* embryos [14]. Moreover, since during normal prenatal pulmonary organogenesis mitotic rates decay in favor of cell differentiation, the higher mitotic rates found in mutant lungs could be a consequence of their delayed differentiation. Accordingly, increased cell proliferation has also been described in lungs of different mouse mutants born with immature lungs, such as in *T1α* and *Crh* gene knockouts [83], [84].

Since lack of IGF1 during lung development causes a general reduction in transcripts of target genes, and a similar result was reported in cochleae of these mice [80], it is possible that IGF1 acts as a general transcriptomic activator during mouse organogenesis. Transcriptome changes found in E18.5 *Igf1^−/−^* lungs could be considered a consequence of their developmental delay. However, this does not seem to be the case because the changes described herein are highly discrepant from transcriptomic profiles reported by Xu et al. at different stages of normal perinatal mouse lung maturation, where they describe major changes in molecular regulators such as FoxM1, Plk1, STATS, EGFR and Notch [79], none of which were observed in the *Igf1^−/−^* lung profile. *Igf1^−/−^* lung transcriptomic analysis revealed changes in networks of biological functions or molecular pathways of functionally related genes. Some of these networks were expected either in light of the lung phenotype or due to previously described IGF1 actions, e.g. in lung development and maturation, cell adhesion, vasculogenesis and MAP kinase signaling. However, others could be identified as potential novel IGF1 target pathways involved in mouse organogenesis, such as genes related to immunity/defense and Wnt signaling pathways, although a recent publication by Ghosh *et al.* suggests that IGF1 indeed promotes alveolar epithelium differentiation through the activation of a non-canonical Wnt pathway [85]. The abundance of differentially expressed genes in the transcription and extracellular regulatory factors category further supports a role for IGF1 as a general regulator of prenatal lung development. However, given that the analysis in the cochlea also rendered genes for specific cochlear maturation and cell differentiation, with only three genes in common to both databases (*Itgav*, *Slc4a1* and *Usp12*) [80], this would further indicate that IGF1 acts with a more tissue specific regulatory role during organogenesis.

E18.5 *Igf1^−/−^* lungs lacking IGF1 showed a general reduction or alteration in ECM deposition, and conversely, addition of IGF1 to explanted lungs elicited a strong increase in laminin expression, which corresponded to their improved epithelial maturation. Accordingly, IGF1 was previously found to stimulate laminin, collagen and other ECM component expressions in different cell types [86]–[88] and *vice versa*: IGF1 expression in type II pneumocytes was considered to be responsible for increased levels of elastin in lungs of FGFR3/4 double mutant mice [89]. The relevance of ECM proteins in lung organogenesis is reflected by impaired lung alveolarization in mutant mice deficient in laminin α5 or elastin [90], [91]. ECM alterations were further supported by a reduction in mRNA expression of genes involved in molecular pathways that regulate cell adhesion, focal adhesion, tight junctions, ECM-receptor interactions (e.g. *Col4a4*, *Eln*, *Fn1*, *Itgb6*, *Icam1*, *Plat1* and *Serpine1*), and the matricellular proteins *Cyr61* and *Ctgf*. Accordingly, the presence of collagen IV in lung basal membranes is required for assembly of intermolecular collagen fibers and proper embryonic lung morphogenesis [92], [93]. Down-regulation of *Fn1* and integrins (e.g. *Itgb6*), components of focal adhesions, indicates alterations in cell-to-cell and cell-to-ECM interactions in the lung. Hence, *Fn1* and *Itgb6* deficiencies in mice cause defects in lung branching morphogenesis and lung emphysema, respectively, and contribute to maturation and organization of alveolar architecture [39], [58]. Finally, matricellular proteins *Cyr61* and *Ctgf* are themselves inducers of ECM accumulation [66].

Our data demonstrate that the lack of IGF1 alters the expression of multiple genes and gene pathways responsible for differentiation of the three foremost relevant cell compartments in the lung, namely epithelium, mesenchyme and endothelium. Thus, we found repressed genes considered to be cell-specific markers or regulators of epithelial maturation, such as *Aqp5*, *Icam1*, *Scgb3a1*, *Fgf18*, *Nfib*, *Klf2*, *Ctgf* and *Cyr61* [17], [32], smooth muscle and vascular development, e.g. actin, Actin regulator (*Phactr*), *Eln*, *Vegfa*, *Flt1/VegfR1*, *Klf2*, *Egr1*, *Ctgf* and *Cyr61* [29], [94]–[97]. Consequently, IGFs signaling has previously been implicated in lung epithelial maturation, vascularization and modulating cellular responses in vascular smooth muscle cells and fibroblasts of pulmonary origin [10], [14], [23], [28], [98]. Altogether, our data clearly demonstrate that lack of IGF1 alters the expression of multiple genes and gene pathways responsible for epithelial, mesenchymal and endothelial lung compartments differentiation.

Our attention was also drawn to the low mRNA levels of immediate-early response genes, such as *Fos*, *Jun*, *Egr1* and *Nr4a1/Nur77* transcription factors and *Cyr61* and *Ctgf* matricellular proteins in *Igf1^−/−^* lungs. It is possible that these genes could decrease their levels in an IGF1-independent direct action, e.g. as a result of the 20 minute period of normal breathing performed by the wild-type embryos. Accordingly, lungs from mouse mutants with impaired breathing capabilities also show altered transcriptomic levels of these immediate-early response genes [51], [99]. However, the fact that IGF1 was reported to specifically induce many of these genes in other contexts would also support the idea that changes in expression of such genes in the lung is dependent on IGF1 signaling [100].

Detailed analysis of Nfib and Klf2 transcription factors and Ctgf and Cyr61 matricellular factors indicate that they are mediators of IGF1 during lung organogenesis. Accordingly, Nfib, Klf2 and Ctgf-deficient mice show delayed pulmonary development with alterations in distal structures [63], [68], [101]. It is relevant to note that the lung phenotype of *Ctgf^−/−^* mice closely resembles that observed in *Igf1^−/−^* lungs, and interestingly, they show diminished levels of IGF1 [68]. Conversely, fetal lungs stimulated to grow by tracheal occlusion show an increased expression of both Ctgf and IGF1 proteins [102], data that strongly support a possible cross-regulation of expression between both proteins during lung development. Furthermore, Ctgf was initially categorized as a low affinity IGFBP, named IGFBP8, due to the presence on its sequence of an IGFBP domain [67]. In a similar context, Cyr61 is a protein structurally related to Ctgf, that it was first named IGFBP10 [67]. Interestingly, the expression levels of these four genes in the highly proliferative *Igf1^−/−^* lungs correlate with their changes in lung cancer. Thus, whereas Nfib was reported as an oncogene in the lung due to its high expression in lung tumors, Klf2, Cyr61 and Ctgf were considered tumor suppressor genes due to their reduced levels [103]–[107]. IGF1 added to explanted lungs induced maturation of distal lung epithelial cells as supposed, however it did not cause the expected expression changes in all the regulatory genes analyzed. Exact reasons for these dissimilar results are unknown, although the fact that *in vitro* cultured explants do not completely mirror the physiological, cellular and molecular events that occur in the lungs *in vivo* should be considered. Thus, the lack of blood supply, and therefore inadequate gas and nutrient perfusion, may incite cell necrosis, among other alterations, that could alter the normal lung cell differentiation program and therefore confound the results. However, in the same scenario, IGF1R expression behaves as expected and serves as a good experimental control gene: its expression increases in the *Igf1^−/−^* explants, probably to compensate for the IGF1 deficit, and its expression is repressed after addition of exogenous growth factor, a similar effect as that described in cultured cell lines [108]. Based on our results from *in vivo* and *ex vivo* experiments, we conclude that IGF1 induces distal lung differentiation by differentially modulating the expression of Nfib, Klf2, Cyr61 and Ctgf. In order to elucidate the molecular mechanisms underlying their expression interdependence, further studies will be required.

To our surprise, *Igf1^−/−^* mice also showed diverse symptoms of lung inflammation. Interestingly, Sanchez-Calderon *et al.* reported a similar effect in the cochlea [80]. Since IGFs are not commonly directly involved in these processes, we surmise that alterations in these gene pathways could be an indirect consequence of IGF1 deficiency disrupting lung homeostasis. A feasible cause of pulmonary inflammation could be reduction in cellular adhesion, as it was described in other systems. Thus, the p120-catenin (a regulator of adherent junctions) conditional mutation in skin triggers a cascade of pro-inflammatory responses that activate ERK1/2 signaling, which finally affects immune homeostasis [109]. In a similar way, loss of *Itgb6* in the lung causes emphysema, mediated by TGF-ß activation and an accumulation of lymphocytes and neutrophils [39]. Deregulation in immune responses has been associated with disturbances in IGF1 and IGF1R in typical neonatal pulmonary diseases, such as respiratory distress syndrome and bronchopulmonary dysplasia [8], [9]. Interestingly, both human diseases, which are characterized by pulmonary immaturity, undifferentiated alveoli with the presence of hyaline membrane and atelectasis, dilated capillaries immersed in the mesenchyme and distorted ECM deposition [110], are correspondingly modeled by prenatal lung phenotype of *Igf1^−/−^* prenatal mouse mutants. A thorough understanding of the molecular mechanisms controlling prenatal lung maturation will help to elucidate pathogeneses of lung diseases associated with preterm birth, providing new potential therapeutic and diagnostic tools to treat pulmonary diseases in infants.

In conclusion, we demonstrate that IGF1 acts as a crucial growth factor with cell-specific roles during embryonic mouse pulmonary development, affecting proliferation during the early stages of development, especially differentiation, adhesion and immunity in prenatal stages. In addition, our results revealed gene networks and novel potential regulatory target gene mediators of IGF1 action on epithelial, mesenchymal and vascular cells in the distal lung of prenatal mouse fetuses. Future studies aimed at further analysis of IGF1 implication on cell signaling and gene regulation in specific pulmonary cell types will be necessary to better understand its function during lung organogenesis.

## Materials and Methods

### Ethics statement

All experiments and animal procedures were carried out in accordance with the Guidelines laid down by the European Communities Council Directive of 24 November 1986 (86/609/EEC) and were revised and approved by the Bioethics Committees of the University of Salamanca and CIBIR (Logroño).

### Mice, embryos, histology and collagen determination

Previously generated *Igf1* mutant mice [11] were backcrossed for at least seven generations into a C57Bl/6J genetic background. Details on mice genotyping, embryo manipulation, histological techniques and collagen determinations are available in *Methods S1* and published elsewhere [23], [24]. For 5-bromo 2-deoxyuridine (BrdU) labeling, 0.1 mg/g body mass of BrdU (Roche, Mannheim, Germany) was administered to pregnant females by intraperitoneal injection. Injected animals were sacrificed 1 h later and embryos processed for paraffin embedding.

### Lung fluorescein angiograms and morphometry of blood vessel diameter

The left ventricle of E18.5 anesthetized embryos was cannulated and injected with 50 µL of 20 mg/mL FITC-dextran (2,000 MW) (Sigma) in PBS. After 10 min, lungs were dissected, fixed overnight at 4°C in 4% paraformaldehyde in PBS, washed in PBS and either directly whole-mounted in anti-fading medium or embedded in OCT medium (Miles Inc.), frozen in 2-methyl butane and kept at −70°C. 10 µm cryostat sections were obtained and mounted on glass slides for immunostaining. Whole-mounted lungs were observed by confocal microscopy and three-dimensional projections of 14 consecutive 1 µm thick images were obtained using the LSM software package (Carl Zeiss, Oberkochen, Germany). The blood vessel area was quantified using OpenLab® Software (Improvision, Coventry, UK) on sections immunostained for laminin and counter-stained with DAPI. At least 25 vessels from each sample on selected 40× fields located at the distal part of the lung were measured. Statistical analyses were performed using the Mann-Whitney *U*-test.

### Lung organ culture

E12.5 lung explants were placed on 1 µm polycarbonate filters (Whatmann, Göttingen, Germany) over stainless-steel grids and were cultured at the air-medium interface in IMEM defined medium (GIBCO-Invitrogen, Paisley, UK) supplemented with 50 µg/mL apo-transferrin (Sigma) and 50 units/µg penicillin/streptomycin [111], [112]. E16.5 lung explants were cultured in the same manner, but using DMEM/F12 defined medium (GIBCO), supplemented with 2 mM glutamine, 100 mg/mL ascorbic acid (Sigma), 50 units/µg penicillin/streptomycin [77], [113] and, when indicated, treated with 100 ng/mL rhIGF1 (R&D). Cultures were maintained up to 96 h in a 5% CO_2_ incubator, and monitored and photographed using an inverted microscope. Cellular proliferation in E12.5 explants was determined by incorporation of BrdU (Roche), following the manufacturer's instructions by adding 10 µM BrdU to the medium 1 h before stopping the culture. For whole-mount BrdU staining, lung explants were fixed in cold methanol, treated with 2 N HCl, and neutralized before immuno-staining with a mouse monoclonal antibody to BrdU (Roche). After incubation with a secondary antibody Cy3-anti-mouse IgG (Jackson Immuno Research; 1∶400 dilution) and counterstaining with Sytox (250 nM; Molecular Probes) lungs were mounted and examined by confocal microscopy. For immuno-histochemical analyses on sections, E12.5 lung explants were fixed and embedded in 7% gelatin (BioRad) and 15% sucrose in PBS. E16.5 lung explants were directly embedded in OCT medium (Miles Inc.), frozen in melting 2-methyl butane and kept at −70°C until used. 10 µm cryostat sections were mounted on glass slides and either analyzed with haematoxylin-eosin or immuno-stained for BrdU.

### RNA isolation, cRNA synthesis and microarray hybridization

Total RNA from homogenized E18.5 lungs was purified using Trizol Reagent® (Invitrogen, Carlsbad, CA) following the manufacturer's instructions, treated with 1 U/µL RNase-free DNase (Promega), purified through RNeasy columns (Qiagen, Valencia, CA) and quantified with RNA NanoLab® Chips (Agilent Technologies, Palo Alto, CA). RNA was then used to synthesize complementary RNA (cRNA) probes for hybridization to GeneChip high-density mouse ‘whole genome’ (MOE430A2.0) oligonucleotide microarrays from Affymetrix (Affymetrix Inc., Santa Clara, CA) according to protocols described in Gene Expression Analysis Technical Manual (http://www.affymetrix.com). Briefly, 5 µg of total RNA obtained from three *Igf1^+/+^* and three *Igf1^−/−^* mouse lungs were separately reverse-transcribed into double-stranded complementary DNA (cDNA), using the SuperScript Choice System for DNA Synthesis (Invitrogen). cDNA was then used as a template to synthesize cRNA by *in vitro* transcription with the incorporation of biotinylated nucleotides (Enzo Diagnostics, Farmingdale, NY, USA). Labeled cRNAs were fragmented and hybridized to six Affymetrix GeneChip 430A2.0 Arrays, using the GeneChip Fluidics Station 450 (Affymetrix). Hybridized arrays were stained with streptavidin–phycoerythrin, according to the manufacturer's protocols. Finally, the stained arrays were scanned in a GeneChip Scanner 3000 (Hewlett Packard, Palo Alto, CA, USA). Raw data of the expression arrays were submitted to Gene Expression Omnibus (http://www.ncbi.nlm.nih.gov/geo/) with accession number GSE17157.

### Bioinformatics microarray data analysis: normalization, differential gene expression and biological classification

The RMA algorithm [114] was used for background correction and normalization of fluorescent hybridization signals of the microarrays as described elsewhere [115]. Bioconductor and R were used as computational tools (www.bioconductor.org) to apply RMA to the data set of 12 microarray hybridizations. In addition to the six microarrays hybridized with lung-derived RNA (three *Igf1^+/+^* and three *Igf1^−/−^*), six microarrays hybridized with cochlear RNA (three *Igf1^+/+^* and three *Igf1^−/−^*) were included in the analysis to normalize the hybridization signals [80]. Cochlear RNA was obtained from the same animals or their siblings and its purification, the synthesis of cRNA and microarray hybridization were performed in parallel with lung RNA [80]. After quantifying the expression level of each probe-set in all microarrays, the SAM algorithm [116] was used to identify probe-sets displaying significant differential expression when comparing the three *Igf1^−/−^* lung samples to their respective three lung controls (*Igf1^+/+^*). The method calculates the type I error or the number of expected false positives using the calculation of the *false discovery rate* (FDR parameter) [115]. In this report, two different levels of FDR cut-off values were used: FDR = 0.20 (p<0.00045; 640 probe-sets) for high-throughput bioinformatics analysis and FDR = 0.10 (p<0.00900; 63 probe-sets) to identify highly relevant IGF1 target genes. The 640 differentially expressed probe-sets (FDR<0.20) were functionally analyzed with three different software tools. Biological functions were determined with the *FatiGo+* application (http://www.ncbi.nlm.nih.gov/pubmed/17478504), based on *GO* gene (*Gene Ontology*) annotations. Over- and under-expressed genes were compared, and those functions with *p*<0.1 (Fisher's exact test) were considered significant [117]. Molecular pathways were analyzed with *GeneCodis* software (http://www.ncbi.nlm.nih.gov/pubmed/19465387), which uses *KEGG* (*Kyoto Encyclopedia of Genes and Genomes*) ontological terms. The list of probe-sets was compared against the whole genome with a hypergeometric test, considering those molecular pathways with *p*<0.1 as significantly relevant [118]. Functional biological interactions were determined using the *Ingenuity Pathways Analysis®*application (http://analysis.ingenuity.com/pa), a web-based software program that identifies molecular networks by relating each gene entry to a comprehensive database of known physical, transcriptional interactions reported for ≈8000 mouse proteins.

### Real-time Quantitative RT-PCR

Real-time quantitative (q) RT-PCR was carried out with SYBR® Green Technology (iScript™ One-Step RT-PCR, Bio-Rad, Hercules, CA) as previously described [23]. Primers used are listed in *Table S6*. Amplification of *ß-2-microglobulin* was used as an internal control to normalize gene expression data [119], except for the *Igf*-system genes in Fig. 6A, where *Gapdh* was instead used.

### Immunohistochemistry and Western blotting

See *Methods S1* for details. Primary antibodies used in immunohistochemistry and Western blots, including their source, dilution, reference and manufacturer, are listed in *Table S7*.

## Supporting Information

Figure S1 **Alterations in cell proliferation and histology of distal parenchyma caused by IGF1-deficiency during development of prenatal mouse lungs.** (**A–B**) Immuno-staining for PCNA in representative paraffin cross-sections shows increased numbers of stained cells (arrows) in E18.5 *Igf1^−/−^* lungs (−/−) in (**B**), when compared with wild-type littermates (+/+) in (**A**). (**C–D**) Haematoxylin-eosin stained cross-sections of E18.5 lungs showing histological alterations in the distal parenchyma. Normal lungs (**C**) show expanded saccular spaces, well-defined thin septa (s) and red cells in capillaries (stained red) mainly lining parenchymal septa, whereas *Igf1*-null lungs (**D**) display reduced air spaces (asterisk) and not well-defined septa, presence of hyaline membranes (arrowhead), and capillaries immersed in the abundant mesenchyme (arrows). These results obtained from mice with a C57Bl/6J background resemble previous data obtained from mutant mice with an out-bred background (Moreno-Barriuso *et al. Dev Dyn* 235: 2040, 2003). a, saccular space; s, septum. Scale bar in D: 20 µm in A–B and 10 µm in C–D.
(DOC)

Figure S2 **Statistical identification of differentially expressed genes in lungs of Igf1-null E18.5 embryos.** Graphical display of statistical analysis performed to identify genes undergoing significant changes of expression in Igf1^−/−^ lungs compared to normal Igf1^+/+^. Two different levels of false discovery rate (FDR) stringency were used. (**A**) Establishing an FDR<0.20 (|Δ(i)|≥2.136; p<0.00090), 640 probe-sets, corresponding to 566 different genes, were identified in the Igf1^−/−^ lungs. Of those, 209 probe-sets were found up-regulated (33%) and 431 down-regulated (67%) (See list in Table S1). (**B**) Considering FDR<0.10 (|Δ(i)|>3.800; p<0.00045), 62 probe-sets (59 genes) were identified as highly relevant IGF1 target genes (See additional information in Table S4). Individual plots were generated by significant analysis of microarrays algorithm (SAM)-contrasting three independent microarray hybridizations, performed with RNA obtained from lungs of three mice of each genotype (Igf1^+/+^ and Igf1^−/−^). Statistically significant gene expression changes occurring between Igf1-null and control lungs were identified using the SAM algorithm (Tusher et al. Proc Natl Acad Sci U S A **98**:5116, 2001). In this analysis six additional microarrays hybridized with cochlear RNA (three Igf1^+/+^ and three Igf1^−/−^), obtained from the same mice or their littermates and hybridized in parallel, were included for background correction and normalization of hybridization. Differential expression for a given gene probe-set is quantitated by Δ(i), measuring the distance of the spot representing its expression value to the no-change diagonal. Green dots identify probe-sets presenting significant alterations of expression, depending on the FDR limit cut-off. Black dots remaining close to the diagonal represent probe-sets whose expression level does not show significant change in Igf1-nulls relative to their controls.
(DOC)

Figure S3 **Network of the functional interactions among the identified genes with differential expression using the Ingenuity Pathways Analysis® database and organized according to their sub-cellular localization.** The analysis included differential expressed genes found in E18.5 Igf1^−/−^ lungs with FDR<0.20, using their functional relations and annotations in the database. Genes in nodes are color-coded in red (up-regulated) or green (down-regulated). The displayed network with 68 genes was generated by fusion of two highly significant networks consisting of 35 and 33 genes respectively, and considering only direct relations between genes according to their Ingenuity annotations. Note the abundance of transcription factors networks and extracellular space proteins, among them IGF1.
(DOC)

Figure S4 **Immunohystochemical staining for Nfib in crossections of lung explants cultured ex vivo.** E16.5 Igf1^+/+^ (A and C) and Igf1^−/−^ (B and D) lung lobes were explanted and cultured in defined medium for 96 h in absence (A–B) or presence (C–D) of recombinant IGF1 (100 ng/mL). Note the high proportion of Nfib-positive mesenchymal cells aligning under epithelial cells of the saccular spaces in IGF1-treated samples of both genotypes (arrows in C and D), effect that is better noticed in Igf1^−/−^ explants (D), and less clear in non-treated explants of both genotypes (A and B). as, airway space; s, septum. Scale bar: 20 µm.
(DOC)

Table S1 **Genes differentially expressed in microarrays of the E18.5 Igf1^−/−^ lungs.** ^a^ List of probe sets found with significant differential expression (FDR<0.20) in Igf1^−/−^ lungs and ordered according to decreasing absolute Δ(i) value. The first 63 probe-sets are functionally tabulated in manuscript Table S4. ^b^ Over-expressed genes/probe-sets are shown in red and repressed genes/probe-sets in green. ^c^ p value obtained after applying the SAM algoritm. ^d^ R fold change relative to the logarithm scale. Corresponds to the n value in 2^n^. ^e^ X, is the total fold change calculated as antilog_2_ of R.
(XLS)

Table S2 **Biological functions based on GO annotations, and the assigned deregulated genes, found with significant changes with the FatyGO+ bioinformatic tool in the differentially expressed genes of Igf1^−/−^ lungs (FDR<0.20) and represented in** **Figure 4A** **.**
(DOC)

Table S3 **Biological functions based on KEGG annotations and the assigned deregulated genes, found with significant changes by the GeneCodis bioinformatic tool in the differentially expressed genes of Igf1^−/−^ lungs (FDR<0.20) and represented in** **Figure 4B** **.**
(DOC)

Table S4 **Genes with up-regulated and down-regulated expression in lungs of E18.5 Igf1^−/−^ embryos with FDR<0.10, as listed in Table S1.** ^a^ Functional assignments given by Gene Ontology (GO in NCBI database) and as described in the literature (see references in text). ^b^ Affimetrix probe-set identification. One asterisk (*) marks additional probe-sets for a given gene found with FDR<0.10. ^c^ Δ(i) is a parameter measuring the statistical distance separating the calculated expression value of each gene probe-set from the non-change diagonal plot. ^d^ R fold is the log_2_ value of the fold change in overexpression (up-regulated in Igf1^−/−^) or repression (down-regulated in Igf1^−/−^) of probe-sets in the collection of microarrays. All values are highly significant (p<0.001), as specified in Table S1and determined using the SAM algorithm. ^e^ qRT-PCR mRNA mean fold change in Igf1^−/−^ respect to Igf1^+/+^ lungs using ß2-microglobin/Arbp as internal control for normalization (n = 4 per genotype, in different embryonic lung RNA samples than those used in microarray analyses). All values are highly significant between genotypes (p<0.001; Mann-Whitney U test). * Second probe-set for a given gene with altered expression.
(DOC)

Table S5 **Data on additional selected repressed genes found in the microarrays with FDR between 0.10 and 0.20 ^a^ which expression was corroborated by qRT-PCR.** ^a^ Additional information on microarray data for these genes is shown in Table S1 under an FDR (False Discovery Rate) between 0.10 and 0.20. ^b^ Functional assignments given by Gene Ontology (GO in NCBI database) and as described in the literature. ^c^ Affimetrix probe-set identification. The asterisk (*) marks additional gene probe-sets for a given gene (Listed in Table S1). ^d^ Δ(i) is a parameter measuring the statistical distance separating the calculated expression value of each gene probe set from the non-change diagonal plot. ^e^ R fold is the log_2_ value of the fold change measuring the repression of the probe sets in the collection of microarrays respect to Igf1^−/−^. All values are significant (p<0.001 for Icam1 and Atf3; p<0.01 for Fn1), as specified in Table S1. ^f^ qRT-PCR fold change in mRNA levels of Igf1^−/−^ lungs respect to Igf1^+/+^controls, using ß2-microglobin/Arbp as internal control for normalization (n = 4 per genotype, in different embryonic lung RNA samples than those used in microarray analyses). All values are significant between genotypes (p<0.01; Mann-Whitney U test). References: [1] Sakai T, Larsen M, Yamada KM (2003). Fibronectin requirement in branching morphogenesis. Nature 423: 876–881. [2] Williams MC (2003). Alveolar type I cells: molecular phenotype and development. Annu Rev Physiol 65: 669–695. [3] Akram A, Han B, Masoom H, Peng C, Lam E, Litvack ML, Bai X, Shan Y, Hai T, Batt J, Slutsky AS, Zhang H, Kuebler WM, Haitsma JJ, Liu M, dos Santos CC (2010). Activating transcription factor 3 confers protection against ventilator-induced lung injury. Am J Respir Crit Care Med. 182:489–500.
(DOC)

Table S6 **Gene Bank accession number and sequence of primers used in qRT-PCR.**
(DOC)

Table S7 **Source, dilution, reference and manufacturer of primary antibodies used in immunohistochemical stainings and Western blots.**
(DOC)

Methods S1 **Mice, histology and immunodetection.**
(DOC)
