# Supplementary figures and images for: Transcriptome Analysis in Prenatal IGF1-Deficient Mice Identifies Molecular Pathways and Target Genes Involved in Distal Lung Differentiation
Source: PLoS One. 2013 Dec 31;8(12):e83028. doi: 10.1371/journal.pone.0083028 (PMC3877002; doi:10.1371/journal.pone.0083028)

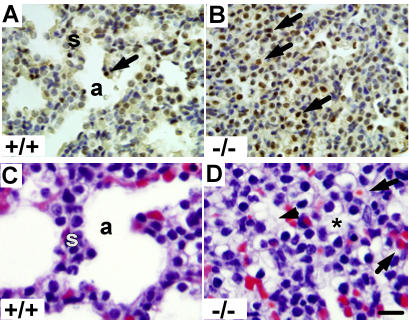

Supplement: Figure S1 — Alterations in cell proliferation and histology of distal parenchyma caused by IGF1-deficiency during development of prenatal mouse lungs. (A–B) Immuno-staining for PCNA in representative paraffin cross-sections shows increased numbers of stained cells (arrows) in E18.5 Igf1−/− lungs (−/−) in (B), when compared with wild-type littermates (+/+) in (A). (C–D) Haematoxylin-eosin stained cross-sections of E18.5 lungs showing histological alterations in the distal parenchyma. Normal lungs (C) show expanded saccular spaces, well-defined thin septa (s) and red cells in capillaries (stained red) mainly lining parenchymal septa, whereas Igf1-null lungs (D) display reduced air spaces (asterisk) and not well-defined septa, presence of hyaline membranes (arrowhead), and capillaries immersed in the abundant mesenchyme (arrows). These results obtained from mice with a C57Bl/6J background resemble previous data obtained from mutant mice with an out-bred background (Moreno-Barriuso et al. Dev Dyn 235: 2040, 2003). a, saccular space; s, septum. Scale bar in D: 20 µm in A–B and 10 µm in C–D. (DOC) [file pone.0083028.s001.doc]

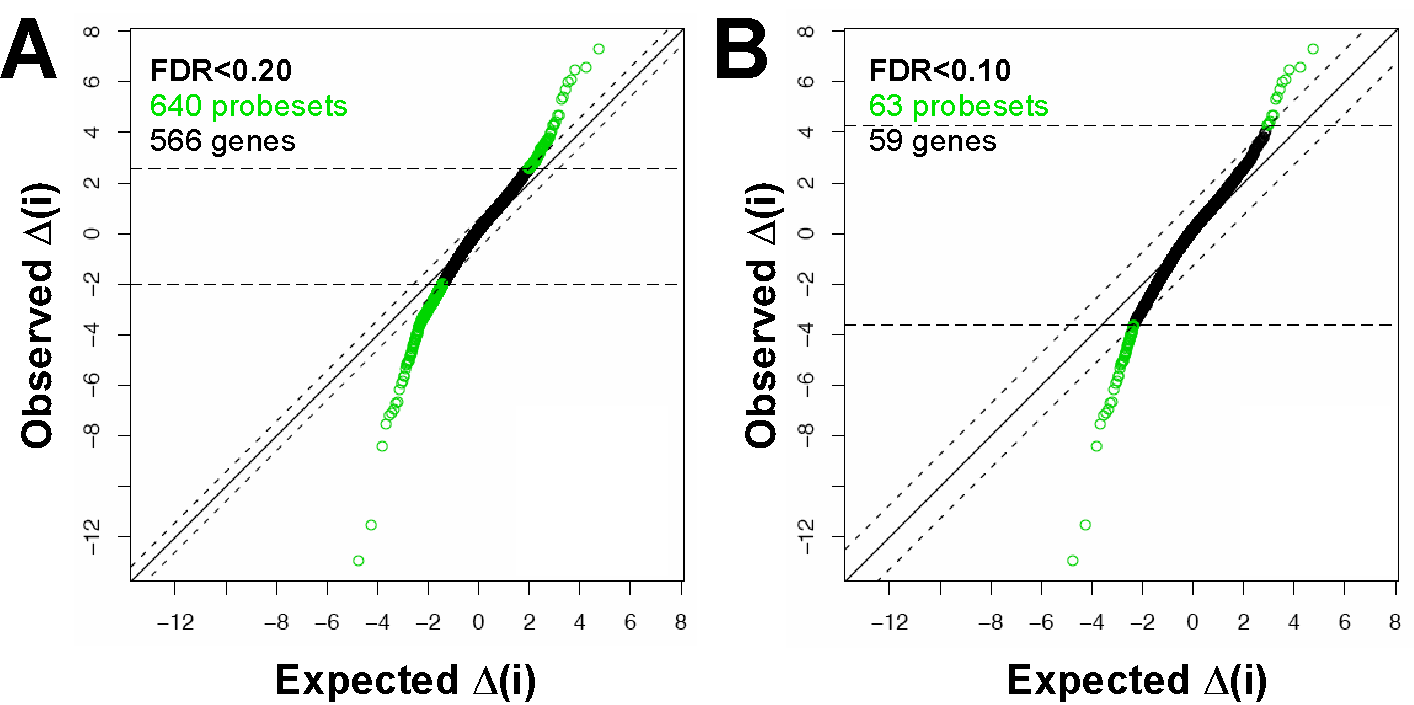

Supplement: Figure S2 — Statistical identification of differentially expressed genes in lungs of Igf1-null E18.5 embryos. Graphical display of statistical analysis performed to identify genes undergoing significant changes of expression in Igf1−/− lungs compared to normal Igf1+/+. Two different levels of false discovery rate (FDR) stringency were used. (A) Establishing an FDR<0.20 (|Δ(i)|≥2.136; p<0.00090), 640 probe-sets, corresponding to 566 different genes, were identified in the Igf1−/− lungs. Of those, 209 probe-sets were found up-regulated (33%) and 431 down-regulated (67%) (See list in Table S1). (B) Considering FDR<0.10 (|Δ(i)|>3.800; p<0.00045), 62 probe-sets (59 genes) were identified as highly relevant IGF1 target genes (See additional information in Table S4). Individual plots were generated by significant analysis of microarrays algorithm (SAM)-contrasting three independent microarray hybridizations, performed with RNA obtained from lungs of three mice of each genotype (Igf1+/+ and Igf1−/−). Statistically significant gene expression changes occurring between Igf1-null and control lungs were identified using the SAM algorithm (Tusher et al. Proc Natl Acad Sci U S A 98:5116, 2001). In this analysis six additional microarrays hybridized with cochlear RNA (three Igf1+/+ and three Igf1−/−), obtained from the same mice or their littermates and hybridized in parallel, were included for background correction and normalization of hybridization. Differential expression for a given gene probe-set is quantitated by Δ(i), measuring the distance of the spot representing its expression value to the no-change diagonal. Green dots identify probe-sets presenting significant alterations of expression, depending on the FDR limit cut-off. Black dots remaining close to the diagonal represent probe-sets whose expression level does not show significant change in Igf1-nulls relative to their controls. (DOC) [file pone.0083028.s002.doc]
