# Supplementary material for: Transcriptome Analysis in Prenatal IGF1-Deficient Mice Identifies Molecular Pathways and Target Genes Involved in Distal Lung Differentiation
Source: PLoS One. 2013 Dec 31;8(12):e83028. doi: 10.1371/journal.pone.0083028 (PMC3877002; doi:10.1371/journal.pone.0083028)
Supplement: Table S2 — Biological functions based on GO annotations, and the assigned deregulated genes, found with significant changes with the FatyGO+ bioinformatic tool in the differentially expressed genes of Igf1−/− lungs (FDR<0.20) and represented in Figure 4A . (DOC) [file pone.0083028.s006.doc]

***Table S2*.** Biological functions based on *GO* annotations, and the assigned deregulated genes, found with significant changes with the *FatyGO+* bioinformatic tool in the differentially expressed genes of *Igf1^-/-^* lungs (FDR<0.20) and represented in Figure 4A.

| **Biological functions** | **Up-regulated genes** |  | **Down-regulated genes** |
| --- | --- | --- | --- |
| ***Vascular development*** | - |  | *Vegfa, Serpine1, Gna13, Elk3,*  *Ctgf, Egfl7, Thbs1, Flt1,*  *Hbegf, Vegfc, Cyr61* |
| ***Immune, defense and inflammatory response*** | *Ggtla1, Igh-6, Ngp, Mcpt6,*  *C1qb, Fcer1g, Fcgr1, C1s,*  *H2Aa, H2DMa, Ifi27,*  *Oas1a, Gzma, Fyb* |  | *Gca, Chst2, Tnfsf13, Nfkbiz,*  *Chst1, Pglyrp1, Fn1, Egr1*  *Ccl6, Samhd1, Wwp1, Cxcl7* |
| ***Organ morphogenesis*** | *Tbx5, Nfib, Crkl* |  | *Ndst1, Vegfa, Serpine1, Wnt3a,*  *Taf4a, Gna13, Elk3, Ctgf, Egfl7,*  *Thbs1, Flt1, Hbegf, Wnt7a, Wwp1,*  *Fgf18, Vegfc, Nrp2, Cyr61, Gja1* |
| ***Cell growth*** | *-* |  | *Nedd9, Ctgf, Ube2e3, Prss11*  *Cyr61,* 0610011/04Rik |
| ***Neural development*** | *Lmx1a, Trappc4, Olig1, Plp1,*  *Cnp1, Cdk5, Utg8, Kcnmb4,*  *Cspg5* |  | *Wnt3a, Sema3a, Robo1, S100a6,*  *Pard6b, Slc12a5* |
