# Supplementary material for: Transcriptome Analysis in Prenatal IGF1-Deficient Mice Identifies Molecular Pathways and Target Genes Involved in Distal Lung Differentiation
Source: PLoS One. 2013 Dec 31;8(12):e83028. doi: 10.1371/journal.pone.0083028 (PMC3877002; doi:10.1371/journal.pone.0083028)
Supplement: Table S3 — Biological functions based on KEGG annotations and the assigned deregulated genes, found with significant changes by the GeneCodis bioinformatic tool in the differentially expressed genes of Igf1−/− lungs (FDR<0.20) and represented in Figure 4B . (DOC) [file pone.0083028.s007.doc]

***Table S3*.** Biological functions based on *KEGG* annotations and the assigned deregulated genes, found with significant changes by the *GeneCodis* bioinformatic tool in the differentially expressed genes of *Igf1^-/-^* lungs (FDR<0.20) and represented in Figure 4B.

| **Metabolic pathway** | **Up-regulated genes** |  | **Down-regulated genes** |
| --- | --- | --- | --- |
| ***MAPK*** | *Elk4, Hspa8, Crkl, Fgf13, Il1r1, Pla2g5, Rac2, Map2k7, Pla2g2e, Cacna1h* |  | *Fos, Jun, JunD1, Nr4a1, Cdc25b, Fgf18, Hspb1, Ppm1a, Dusp1, Rap1b, 1500003o03Rik, Dusp10* |
| ***Wnt*** | *Fzd3, Sfrp1, Rac2, Ccnd2* |  | *Fzd4, Wnt3a, Wnt7a, Jun,* 1500003o03Rik, *Nkd1* |
| ***Calcium*** | *Itpka, Mylk2* |  | *Sphk1* |
| ***Focal adhesion*** | *Ccnd2, Crkl, Itga2b, Rac2, Mylk2* |  | *Itga9, Capn2, Col4a4, Flt1, Fn1, Igf1, Itgb6, Jun, Rap1, Thbs1, Vegfa, Vegfc* |
| ***Cell adhesion*** | *H2-Aa, H2-Eb1, HA-Dma, H2-Q7, Selpl, Cldn19, Cldn7* |  | *Itga9, Icam1, Pvrl3* |
| ***ECM-receptor interaction*** | *Itga2b* |  | *Itga9, Fn1, Itgb6, Dag1, Thbs1, Col4a4* |
| ***Tight junctions*** | *Cldn19, Cldn7* |  | *Tjp2, Epb4.1, Pard6b, Mpp5* |
| ***Haematopoietic cell lineage*** | *H2-Eb1, Il1r1, Itga2b, Fcgr1* |  | *Kitl* |
| ***Antigen processing and presentation*** | *H2-Aa, H2-Eb1, HA-Dma, Hspa8* |  | *-* |
| ***Leukocyte transendothelial migration*** | *Rac2, Cldn19, Cldn7* |  | *Icam1, Rap1b* |
| ***B-cell receptor signaling*** | *Rac2* |  | *Fos, Jun,* 1500003o03Rik |
