# Supplementary material for: Transcriptome Analysis in Prenatal IGF1-Deficient Mice Identifies Molecular Pathways and Target Genes Involved in Distal Lung Differentiation
Source: PLoS One. 2013 Dec 31;8(12):e83028. doi: 10.1371/journal.pone.0083028 (PMC3877002; doi:10.1371/journal.pone.0083028)
Supplement: Table S4 — Genes with up-regulated and down-regulated expression in lungs of E18.5 Igf1−/− embryos with FDR<0.10, as listed in Table S1. a Functional assignments given by Gene Ontology (GO in NCBI database) and as described in the literature (see references in text). b Affimetrix probe-set identification. One asterisk (*) marks additional probe-sets for a given gene found with FDR<0.10. c Δ(i) is a parameter measuring the statistical distance separating the calculated expression value of each gene probe-set from the non-change diagonal plot. d R fold is the log2 value of the fold change in overexpression (up-regulated in Igf1−/−) or repression (down-regulated in Igf1−/−) of probe-sets in the collection of microarrays. All values are highly significant (p<0.001), as specified in Table S1and determined using the SAM algorithm. e qRT-PCR mRNA mean fold change in Igf1−/− respect to Igf1+/+ lungs using ß2-microglobin/Arbp as internal control for normalization (n = 4 per genotype, in different embryonic lung RNA samples than those used in microarray analyses). All values are highly significant between genotypes (p<0.001; Mann-Whitney U test). * Second probe-set for a given gene with altered expression. (DOC) [file pone.0083028.s008.doc]

***Table S4*.** Genes with up-regulated and down-regulated expression in lungs of E18.5 *Igf1 ^-/-^* embryos with FDR <0.10

| ***Functional class ^a^***  Probe ID **^b^** | **∆(i) ^c^** | **Gene Chip**  **fold change ^d^** | **qRT-PCR**  **fold change ^e^** | ***Gene*** | **Gene title** |
| --- | --- | --- | --- | --- | --- |
| **UP-REGULATED GENES** | | |  |  |  |
| ***Immune/defence/inflammation response*** | | |  |  |  |
| 1435290_x_at | 7.30 | 3.64 | 2.73 | *H2-Aa* | Histocompatibility 2 class II antigen A. alpha |
| 1417898_a_at | 4.65 | 4.88 |  | *Gzma* | Granzyme A |
| 1418612_at | 3.81 | 2.85 |  | *Slfn1* | Schlafen 1 |
| ***Lung development and maturation*** | | |  |  |  |
| 1438245_at | 3.93 | 2.62 | 2.50 | *Nfib* | Nuclear factor I/B |
| ***Ribosome associated and protein biosynthesis*** | | |  |  |  |
| 1455904_at | 6.56 | 10.01 | 2.83 | *Gas5* | Growth arrest specific 5 |
| 1438076_at | 6.00 | 4.10 | 2.58 | *Rpl30* | Ribosomal protein L30 |
| 1426958_at | 5.72 | 3.50 |  | *Rps9* | Ribosomal protein S9 |
| 1436384_at | 5.39 | 3.74 |  | *Rps10* | Ribosomal protein S10 |
| 1435655_at | 4.27 | 4.81 | 2.83 | *Rpl12* | Ribosomal protein L12 |
| ***Chaperoning and stress response*** | | |  |  |  |
| 1431182_at | 6.11 | 4.62 | 3.16 | *Hspa8 (Hsc70)* | Heat shock protein 8 |
| 1438064_at | 4.69 | 3.16 |  | *Nsep1 (Yb-1)* | Nuclease sensitive element binding protein 1 |
| 1421416_at | 4.28 | 2.44 |  | *Map2k7 (Jnkk2)* | Mitogen activated protein kinase kinase 7 |
| ***Integral to ER membrane*** | | |  |  |  |
| 1426981_at | 5.33 | 2.97 |  | *Pcsk6 (Pace4)* | Proprotein convertase subtilisin/kexin type 6 |
| 1449104_at | 4.42 | 3.42 |  | *Upk3a* | Uroplakin 3A |
| ***Mitochondrial enzymes*** | | |  |  |  |
| 1459885_s_at | 4.01 | 3.38 |  | *Cox7c* | Cytochrome c oxidase. subunit VIIc |
| 1426959_at | 3.81 | 2.45 |  | *Bdh* | 3-hydroxybutyrate dehydrogenase (heart. mitochondrial) |
| ***Other/Unknown*** | | |  |  |  |
| 1428013_at | 6.50 | 3.22 |  | *6030458C11Rik* | RIKEN cdna 6030458C11 gene |
| 1429207_at | 4.31 | 3.08 |  | *1810009A15Rik* | RIKEN cdna 1810009A15 gene |
| 1455874_at | 4.04 | 2.99 |  | *1810059G22Rik* | RIKEN cdna 1810059G22 gene |
| **DOWN-REGULATED GENES** | | |  |  |  |
| ***Vascular development*** | | |  |  |  |
| 1416039_x_at | -12.94 | 43.59 | >100 | *Cyr61 (Igfbp10, CCN1)* | Cysteine rich protein 61 |
| *1438133_a_at | -11.53 | 68.39 |  |  |  |
| 1416953_at | -5.19 | 3.59 | 10.92 | *Ctgf (Igfbp8, CCN2)* | Connective tissue growth factor |
| 1451959_a_at | -4.31 | 3.52 | 6.51 | *Vegfa* | Vascular endothelial growth factor A |
| 1453128_at | -3.93 | 2.80 |  | *Xlkd1 (Lyve-1)* | Extra cellular link domain-containing 1 |
| ***Lung development and maturation*** | | |  |  |  |
| 1448890_at | -8.40 | 4.22 | 5.42 | *Klf2 (Lklf)* | Kruppel-like factor 2 (lung) |
| 1449545_at | -5.02 | 2.95 | 8.72 | *Fgf18* | Fibroblast growth factor 18 |
| 1418818_at | -4.48 | 4.65 |  | *Aqp5* | Aquaporin 5 |
| ***MAP kinase signaling pathway/Immediate early response/Stress*** | | | | |  |
| 1423100_at | -7.12 | >100 | <100 | *Fos* | FBJ osteosarcoma oncogene |
| 1416505_at | -6.94 | 91.42 | 7.25 | *Nr4a1 (Nur77)* | Nuclear receptor subfamily 4, group A, member 1 |
| 1417409_at | -6.17 | 3.15 | 5.42 | *Jun (AP1)* | Jun oncogene |
| *1448694_at | -4.82 | 3.30 |  |  |  |
| 1448830_at | -5.82 | 8.73 | 7.68 | *Dusp1 (MKP-1)* | Dual specificity phosphatase 1 |
| 1417065_at | -5.63 | 49.01 | 4.51 | *Egr1* | Early growth response 1 |
| ***Calcium metabolism*** | |  |  |  |  |
| 1449166_at | -6.73 | 3.14 | 3.70 | *S100a14* | S100 calcium binding protein A14 |
| 1416601_a_at | -4.74 | 2.83 |  | *Dscr1* | Down syndrome critical region homolog 1 (human) |
| ***Erythropoiesis and eryithrocyte related*** | | |  |  |  |
| 1416464_at | -7.56 | 10.47 | 2.90 | *Slc4a1 (Band3)* | Solute carrier family 4 (anion exchanger), member 1 |
| 1423016_a_at | -6.64 | 9.45 | 3.12 | *Gypa* | Glycophorin A |
| 1449077_at | -5.93 | 7.73 | 5.91 | *Eraf* | Erythroid associated factor |
| ***Antiproliferative & tumor suppressor*** | | |  |  |  |
| 1419699_at | -5.64 | 8.13 | 23.35 | *Scgb3a1 (HIN-1)* | Secretoglobin, family 3A, member 1 |
| 1448272_at | -5.36 | 3.60 | 6.20 | *Btg2 (TIS21)* | B-cell translocation gene 2, anti-proliferative |
| ***Cell adhesion and extracellular matrix*** | | |  |  |  |
| 1415806_at | -5.05 | 3.16 |  | *Plat (tPA)* | Plasminogen activator, tissue |
| 1418511_at | -4.73 | 4.67 |  | *Dpt (Eq-1)* | Dermatopontin |
| 1451537_at | -4.68 | 14.37 |  | *Chi3l1* | Chitinase 3-like 1 |
| 1432281_a_at | -4.23 | 3.97 | 3.00 | *Itgb6* | Integrin beta 6 |
| 1460238_at | -3.98 | 3,00 |  | *Msln* | Mesothelin |
| ***Metabolic enzymes*** | | |  |  |  |
| 1415793_at | -5.06 | 3.02 |  | *Pnpo* | Pyridoxine 5'-phosphate oxidase |
| 1418698_a_at | -4.20 | 3.36 |  | *Fech* | Ferrochelatase |
| 1424400_a_at | -3.99 | 2.97 |  | *Fthfd* | Formyltetrahydrofolate dehydrogenase |
| ***Immune/defence/inflammation response*** | | |  |  |  |
| 1427747_a_at | -4.86 | 3.69 |  | *Lcn2 (NGAL)* | Lipocalin 2 |
| 1415854_at | -4.42 | 3.32 | 2.91 | *Kitl (SCF)* | Kit ligand |
| 1452519_a_at | -4.36 | 3.59 | 2.77 | *Zfp36 (Ttp)* | Zinc finger protein 36 |
| ***Development*** | | |  |  |  |
| 1423367_at | -4.31 | 2.94 | 13.30 | *Wnt7a* | Wingless-related MMTV integration site 7A |
| 1418280_at | -4.00 | 3.42 | 4.00 | *Klf6* | Kruppel-like factor 6 |
| *1427742_a_at | -3.89 | 2.98 |  |  |  |
| ***Other/Unknown*** |  |  |  |  |  |
| 1451425_a_at | -5.16 | 3.86 |  | *Mkrn1* | Makorin. ring finger protein 1 |
| 1417750_a_at | -5.09 | 4.16 |  | *Slc25a37* | Mitochondrial solute carrier protein family 25, member 37 |
| 1427932_s_at | -4.58 | 3.55 |  | *1200016E24Rik* | RIKEN cdna 1200016E24 gene |
| 1416101_a_at | -4.17 | 2.92 |  | *Hist1h1c* | Histone 1, H1c |
| 1418990_at | -4.17 | 4.42 |  | *Ms4a4d* | Membrane-spanning 4-domains. subfamily A. member 4D |
| 1450786_x_at | -4.10 | 2.92 |  | *Pdlim5* | PDZ and LIM domain 5 |
| 1418108_at | -3.98 | 14.91 |  | *Plekhk1* | Pleckstrin homology domain containing. family K member 1 |
| 1424669_at | -3.86 | 3.59 |  | *Zfyve21* | Zinc finger. FYVE domain containing 21 |
