# Supplementary material for: Transcriptome Analysis in Prenatal IGF1-Deficient Mice Identifies Molecular Pathways and Target Genes Involved in Distal Lung Differentiation
Source: PLoS One. 2013 Dec 31;8(12):e83028. doi: 10.1371/journal.pone.0083028 (PMC3877002; doi:10.1371/journal.pone.0083028)
Supplement: Table S5 — Data on additional selected repressed genes found in the microarrays with FDR between 0.10 and 0.20 a which expression was corroborated by qRT-PCR. a Additional information on microarray data for these genes is shown in Table S1 under an FDR (False Discovery Rate) between 0.10 and 0.20. b Functional assignments given by Gene Ontology (GO in NCBI database) and as described in the literature. c Affimetrix probe-set identification. The asterisk (*) marks additional gene probe-sets for a given gene (Listed in Table S1). d Δ(i) is a parameter measuring the statistical distance separating the calculated expression value of each gene probe set from the non-change diagonal plot. e R fold is the log2 value of the fold change measuring the repression of the probe sets in the collection of microarrays respect to Igf1−/−. All values are significant (p<0.001 for Icam1 and Atf3; p<0.01 for Fn1), as specified in Table S1. f qRT-PCR fold change in mRNA levels of Igf1−/− lungs respect to Igf1+/+controls, using ß2-microglobin/Arbp as internal control for normalization (n = 4 per genotype, in different embryonic lung RNA samples than those used in microarray analyses). All values are significant between genotypes (p<0.01; Mann-Whitney U test). References: [1] Sakai T, Larsen M, Yamada KM (2003). Fibronectin requirement in branching morphogenesis. Nature 423: 876–881. [2] Williams MC (2003). Alveolar type I cells: molecular phenotype and development. Annu Rev Physiol 65: 669–695. [3] Akram A, Han B, Masoom H, Peng C, Lam E, Litvack ML, Bai X, Shan Y, Hai T, Batt J, Slutsky AS, Zhang H, Kuebler WM, Haitsma JJ, Liu M, dos Santos CC (2010). Activating transcription factor 3 confers protection against ventilator-induced lung injury. Am J Respir Crit Care Med. 182:489–500. (DOC) [file pone.0083028.s009.doc]

***Table S5*.** Data on additional selected repressed genes found in the microarrays with FDR between 0.10 and 0.20 ^a^ which expression was corroborated by qRT-PCR.

| ***Functional class ^b^***  Probe ID **^c^** | **Δ(i) ^d^** | **Gene Chip**  **fold change ^e^** | **qRT-PCR**  **fold change ^f^** | ***Gene*** | **Gene title** | **References** |
| --- | --- | --- | --- | --- | --- | --- |
| ***Cellular adhesion and extracellular matrix*** | | |  |  |  |  |
| 1426642_at | -2,63 | 3,03 | 17,96 | *Fn1* | Fibronectin 1 | [1] |
| *1437218_at | -2,28 | 2,58 |  |  |  |  |
| ***Type I neumocyte differentiation*** | | |  |  |  |  |
| 1424067_at | -3,53 | 2,96 | 2,90 | *Icam1* | Intercellular adhesion molecule | [2] |
| ***Immediate-early response transcription factor*** | | | | |  |  |
| 1449363_at | -3,25 | 4,46 | 2,77 | *Atf3* | Activating transcription factor 3 | [3] |
