# Supplementary material for: Transcriptome Analysis in Prenatal IGF1-Deficient Mice Identifies Molecular Pathways and Target Genes Involved in Distal Lung Differentiation
Source: PLoS One. 2013 Dec 31;8(12):e83028. doi: 10.1371/journal.pone.0083028 (PMC3877002; doi:10.1371/journal.pone.0083028)
Supplement: Table S6 — Gene Bank accession number and sequence of primers used in qRT-PCR. (DOC) [file pone.0083028.s010.doc]

***Table S6*.** Gene Bank accession number and sequence of primers used in qRT-PCR.

| **Gene** | **Accession No.** | **Forward primer** | **Reverse primer** |
| --- | --- | --- | --- |
| **Aqp-5** | NM_009701 | 5' GGTGGTCATGAATCGGTTCAGC 3' | 5' GTCCTCCTCTGGCTCATATGTG 3' |
| **Arbp** | NM_007475 | 5' GTGTTTGACAACGGCAGCATT 3' | 5' TTGATGATGGAGTGTGGCACC 3' |
| **Atf3** | NM_007498 | 5' CCTGTGGTGACCTACTGCATTG 3' | 5' GGTGAGACAAAGGATGCTCCTG 3' |
| **B2m** | NM_009735 | 5' CCTGGCTCACACTGAATTCACC 3' | 5' TCGATCCCAGTAGACGGTCTTG 3' |
| **Btg2** | NM_007570 | 5' CCAGGGAACTCCAAGTCCTTTC 3' | 5' GCACAGTGACTGAGAACGCTTG 3' |
| **Ctgf** | NM_010217 | 5' TTCTGGCTGCACCAGTGTGAAG 3' | 5' TTGAACTCCACTGGCAGAGTGG 3' |
| **Cyr61** | NM_010516 | 5' TGCAGGATGCTCCAGTGTCAAG 3' | 5' TCACAGTTCTGGTCTGCAGAGG 3' |
| **Dusp1** | NM_013642 | 5' ACTAGTGTGCCTGACAGTGCAG 3' | 5' AGGAAGGACAGGATCTCCACTG 3' |
| **Egr1** | NM_007913 | 5' CTATGAGCACCTGACCACAGAG 3' | 5' TTTATAACTCGTCTCCACCATC 3' |
| **Eraf** | NM_133245 | 5' CCAGCAAGAGCTGAGTACTCTG 3' | 5' GAGGAGGGCAGTGTATTGCTTG 3' |
| **Fgf18** | NM_008005 | 5' GCTCCTAGTGGAGACAGATACC 3' | 5' ACACGCACTCCTTGCTAGTACC 3' |
| **Fn1** | NM_010233 | 5' TCTACCAGTGCGACTCTGACTG 3' | 5' CTGCAGTGCCTCCACTATGATG 3' |
| **Fos** | NM_010234 | 5' GGAATGGTGAAGACCGTGTCAG 3' | 5' CCTCTTCAGGAGATAGCTGCTC 3' |
| **Gapdh** | M_32599 | 5' TCCTGCACCACCAACTGCTTAG 3' | 5' TGGCATGGACTGTGGTCATGAG 3' |
| **Gas5** | NR_002840 | 5' GACTCAGCATGCGTGTTCATGC 3' | 5' TGACTCAGCATGCTCTGCCATG 3' |
| **Gypa** | NM_010369 | 5' AGAGCACAGCTGCTGTGACAAC 3' | 5' ACAGTTGAAGCCACCACAGGAG 3' |
| **H2Aa** | NM_010378 | 5' GACTGTCTGGATGCTTCCTGAG 3' | 5' AGTCGCTTGAGGAGCCTCATTG 3' |
| **Hsp8** | NM_031165 | 5' GCTGGCTGGATAAGAACCAGAC 3' | 5' ACCTGCACTCTGGTACAGCTTG 3' |
| **Icam** | NM_010493 | 5' GCTCGGAGGATCACAAACGAAG 3' | 5' GACCATACAGCACGTGCAGTTC 3' |
| **Itgb6** | NM_021359 | 5' TGAGCAGACTCTGCAAGTGCAG 3' | 5' GATGGTGTTGAGGTCGTCATCC 3' |
| **Igfbp2** | NM_008342 | 5' GGGAGTGCTGGTGTGTGA 3' | 5' CTGCTGGTGTTCGGGATG 3' |
| **Igfbp4** | NM_010517.3 | 5' TGTGAGATTGGATTGTGTGTGT 3' | 5' TAGAGATGGCGGGATAGGAG 3' |
| **Igfbp6** | NM_008344 | 5' AGGAGAGCAAACCCCAAGGA 3' | 5' TGAACAGGATTGGGCCGTATA 3' |
| **Igf1r** | NM_010513 | 5' TGGGAGGGTTTCAGGACA 3' | 5' AGGTAGGCACAGCATTCGTT 3' |
| **Igf2** | NM_010514 | 5' CCTTCGCCTTGTGCTGCAT 3' | 5' ACGGTTGGCACGGCTTGAA 3' |
| **Igf2r** | NM_010515 | 5' AGGCACCAAGATGAAGCAGT 3' | 5' AATGAAGGGGAAGACACAGG 3' |
| **InsrA** | NM_010568.2 | 5' TCCTGAAGGAGCTGGAGGAGT 3' | 5' CTTTCGGGATGGCCTGG 3' |
| **InsrB** | EU_346716 | 5' TCCTGAAGGAGCTGGAGGAGT 3' | 5' TTCGGGATGGCCTACTGTC 3' |
| **Jun** | NM_010591 | 5' ACAGGTGGCACAGCTTAAGCAG 3' | 5' GCAACTGCTGCGTTAGCATGAG 3' |
| **Kitl** | NM_013598 | 5' GTTGTTGGAGCAGAGTGCTTCG 3' | 5' ACGGTGCAAGTAGACTGGACAC 3' |
| **Klf2** | NM_008452 | 5' ACGAGCTTACCCGCCACTACCG 3' | 5' CATGTGTCGCTTCATGTGCAAGG 3' |
| **Klf6** | NM_011803 | 5' CAGAGGACTCTCTGATCAGCTC 3' | 5' GAACTGTCCGAAGACTCACTGC 3' |
| **Nfib** | NM_008687 | 5' GTGTTCAGCCACACCACATCAC 3' | 5' GTCCAGAATCTTGCTCCTGCAC 3' |
| **Nr4a1** | NM_010444 | 5' CTGGTCCTCATCACTGATCGAC 3' | 5' TGTGCTCCTTCAGACAGCTAGC 3' |
| **RpL12** | NM_009076 | 5' GCGATGACATTGCCAAGGCTAC 3' | 5' CCTGTCTGTTCTGGATGGTCAG 3' |
| **RpL30** | NM_009083 | 5' AAGTCTCTGGAGTCGATCAACTC 3' | 5' GTCTGATCATCTTCAGAGTCTGC 3' |
| **S100a14** | NM_025393 | 5' CTGAGCTTCGAGACCTGGTTAC 3' | 5' TCACACTCTTGGCTGCTTCTCC 3' |
| **Scg3a1** | NM_054037 | 5' CTGCTCAGTGACTCTGGTGTTG 3' | 5' GATGAACCTCAGGATGGCCAAG 3' |
| **Slc4a1** | NM_011403 | 5' GCTGCAGCAACTCTCATGACAG 3' | 5' GTCCAGAAAGCTCTCCAGAGAG 3' |
| **Vegfa** | NM_009505 | 5' TGTACCTCCACCATGCCAAGTG 3' | 5' GGACTTCTGCTCTCCTTCTGTC 3' |
| **Vegfa** | NM_009505 | 5' ACCTCCACCATGCCAAGTGGTC 3' | 5' CAATCGGACGGCAGTAGCTTCG 3' |
| **Wnt7a** | NM_009527 | 5' GCTCCTGTACCACTAAGACGTG 3' | 5' TGTCCTTGAGCACGTAGCCTAG 3' |
| **Zfp36** | NM_011756 | 5' GAGCTCTGTCGGACCTACTCAG 3' | 5' GGAGGTAGAACTTGTGGCAGAG 3' |
