# Supplementary material for: Transcriptome Analysis in Prenatal IGF1-Deficient Mice Identifies Molecular Pathways and Target Genes Involved in Distal Lung Differentiation
Source: PLoS One. 2013 Dec 31;8(12):e83028. doi: 10.1371/journal.pone.0083028 (PMC3877002; doi:10.1371/journal.pone.0083028)
Supplement: Table S7 — Source, dilution, reference and manufacturer of primary antibodies used in immunohistochemical stainings and Western blots. (DOC) [file pone.0083028.s011.doc]

***Table S7*.** Source, dilution, reference and manufacturer of primary antibodies used in immunohistochemical stainings and Western blots.

| **Primary antibody** | **Origin** | **Dilution** | **Reference and manufacturer** |
| --- | --- | --- | --- |
| **Immunohistochemistry** |  |  |  |
| Actin, muscle (HHF35) | Mouse monoclonal | None | MU090-UC; Biogenex |
| Actin, α smooth muscle (1A4) | Mouse monoclonal | 1:200 | A5228; Sigma |
| Aqp5 (C-19) | Goat policlonal | 1:50 | sc-9890; Santa Cruz Biotech. Inc. |
| BrdU (BMC 9318) | Mouse monoclonal | 1:20 | Roche Molecular Biochemicals |
| CD31 / PECAM (M20) | Goat policlonal | 1:100 | sc-1506; Santa Cruz Biotech. Inc. |
| CCSP/CC10/Scgb1a1 (T18) | Goat monoclonal | 1:400 | sc-9772; Santa Cruz Biotech. Inc. |
| Cleaved Caspase-3 (5A1) | Rabbit monoclonal | 1:100 | #9664; Cell Signaling Tech., Inc. |
| IGF-IRβ (C-20) | Rabbit polyclonal | 1:50 | sc-713; Santa Cruz Biotech. Inc. |
| IGF2 | Goat polyclonal | 1:25 | AF292; R&D Systems |
| Laminin | Rabbit policlonal | 1:100 | L9393; Sigma |
| Ly6G/6C / Gr1 (RB6-8C5) | Rat monoclonal | 1:100 | 553126; BD Biosciences |
| NFIB/NF1B2 | Rabbit polyclonal | 1:100 | ab11989; Abcam (Cambridge, UK) |
| PCNA (24) | Mouse monoclonal | 1:50 | 610665; BD Biosciences |
| Pro-SPC (M20) | Goat policlonal | 1:100 | sc-7706; Santa Cruz Biotech. Inc. |
| **Western blot** |  |  |  |
| IGF-IRβ (C-20) | Rabbit polyclonal | 1:1,000 | sc-713; Santa Cruz Biotech. Inc. |
| Cyr-61 (H-78) | Rabbit polyclonal | 1:1,000 | sc-13100; Santa Cruz Biotech. Inc. |
| Egr-1 (588) | Rabbit polyclonal | 1:1,000 | sc-110; Santa Cruz Biotech. Inc. |
| Klf-2 | Rabbit polyclonal | 1:1,000 | AB4137; Chemicon Inter. Inc. |
| NFIB/NF1B2 | Rabbit polyclonal | 1:1,000 | ab119989, Abcam (Cambridge, UK) |
| c-jun (D) | Rabbit polyclonal | 1:1,000 | sc-44; Santa Cruz Biotech. Inc. |
| p-SAPK/JNK (Thr202/Tyr204) | Rabbit polyclonal | 1:1,000 | #9251; Cell Signaling Tech., Inc. |
| JNK1/3 (C-17) | Rabbit monoclonal | 1:500 | sc-474; Santa Cruz Biotech. Inc. |
| CD3 (KT3) | Rat monoclonal | 1:200 | CBL1317; Chemicon Inter. Inc. |
| p-AKT (Ser473) | Rabbit monoclonal | 1:1,000 | #9271; Cell Signaling Tech., Inc. |
| AKT | Rabbit polyclonal | 1:1,000 | #9272; Cell Signaling Tech., Inc. |
| p-ERK1/2 (Thr202/Tyr204) | Mouse monoclonal | 1:1,000 | #9106; Cell Signaling Tech., Inc. |
| ERK-2 (C-14) | Rabbit polyclonal | 1:1,000 | sc-154; Santa Cruz Biotech. Inc. |
| p-p38 (Thr180/Tyr182) | Rabbit monoclonal | 1:1,000 | #4631; Cell Signaling Tech., Inc. |
| p38 αMAP Kinase | Rabbit polyclonal | 1:1,000 | #9218; Cell Signaling Tech., Inc. |
| p-STAT-3 (Tyr705) | Rabbit polyclonal | 1:1,000 | #9131; Cell Signaling Tech., Inc. |
| STAT-3 (F-2) | Mouse monoclonal | 1:500 | sc-8019; Santa Cruz Biotech. Inc. |
| Laminin | Rabbit polyclonal | 1:1,000 | L9393; Sigma (St. Louis, MI) |
| β-Tubulin (TUB 2.1) | Mouse monoclonal | 1:20,000 | T4026; Sigma (St. Louis, MI) |
