# Supplementary material for: Transcriptome Analysis in Prenatal IGF1-Deficient Mice Identifies Molecular Pathways and Target Genes Involved in Distal Lung Differentiation
Source: PLoS One. 2013 Dec 31;8(12):e83028. doi: 10.1371/journal.pone.0083028 (PMC3877002; doi:10.1371/journal.pone.0083028)
Supplement: Methods S1 — Mice, histology and immunodetection. (DOC) [file pone.0083028.s012.doc]

**Supporting Methods S1.** Mice, histology and immunodetection.

***Mouse genotyping and embryo collection***

Mice were either genotyped by Southern blot analysis of tail DNA as previously described , or by standard PCR using specific primers for the *Igf1* wild type allele (*Igf1* forward 5’-GTCTAACACCAGCCCATTCTGATT-3’ and *Igf1* reverse 5’-ACTCGATTTCACCCACTCGATCG-3’) that generate a 250 bp amplicon, and the *Igf1* mutant allele (*Neo* forward 5’- GCTTGGGTGGAGAGGCTATTC -3’ and *Neo* reverse 5’- CAAGGTGAGATGACAGGAGATC -3’), that render a 280 bp-long amplicon. For embryo staging, E0.5 was considered the day when formation of a vaginal plug was first observed. E12.5 and E16.5 embryos were obtained and their dissected lungs used for lung organ cultures. E18.5 pups were prematurely delivered by C-section and helped to breath for 20 min under a warming lamp before weighing and lung dissection. A minimum of n=3 animals for each genotype was used in each experiment, as it was specified on each figure legend.

***Histology and determination of collagen and elastic fibers***

E18.5 dissected lungs were weighted and either fresh frozen in liquid nitrogen or OCT fixed overnight by immersion fixatives. After fixation, either in 4% paraformaldehyde in PBS at 4ºC or in buffered formalin at room temperature, samples were embedded in gelatin or paraffin, respectively. 10 μm-thick criostate (OCT or gelatin embedded) or 4 μm-thick microtome (paraffin embedded) sections were deposited onto slides and then subjected to either routine histological or immunohistochemical staining. Collagen fibers in E18.5 lungs were visualized with a Sirius red stain. Collagen contents was assessed using Collagen Assay kit (Sircol™, Biocolor Ltd., Northern Ireland, UK), following manufacturer instructions. Elastic fibres and collagen were stained with Orcein and Sirius Red solutions (Sigma), respectively.

***Immunohistochemistry***

Paraffin (4 μm) and gelatin or OCT crio-sections (10 μm) were rehydrated in PBS by standard methods, blocked and immunostained as described . Primary antibody used, including their source, dilution, reference and manufacturer, are listed in *Table S7***.** Antigen retrieval was performed, except in samples to determine Actin (clone HHF35) and Ly-6G/6C(Gr1). For PCNA, laminin and Nfib staining, sections were incubated at 95ºC in 10 mM citrate buffer (pH 6.0) for 15 min, followed by a 5 min cool down in the same buffer and 3 successive 5 min incubation at 95ºC. For IGF1R, Aqp5, CD31, CCSP, Pro-SPC and SMA (clone1A1) immunohistochemistry, antigen retrieval was performed with slides immersed in 10 mM citrate buffer (pH 6.0) in a pressure cooker, heating for 3 minutes after reaching full pressure, and followed by a 5 min cool down in the same buffer. For IGF2 immuno-staining antigen retrieval was performed with slides immersed in a boiling solution of 1 mM EDTA (pH 9.0) for 25 minutes. For BrdU determination sections were treated with 2N HCl for 1 h, neutralized with PBS 6 times/10 min each. Samples to be developed with peroxidase were treated with 3% hydrogen peroxide (Sigma). Sections were then blocked and incubated with primary antibodies (*Table S7*). After washing, sections were incubated for 2 h., either with biotin- or fluorochrome-conjugated secondary antibodies. Host specific biotinilated antibodies (Vector Laboratories) were visualized with avidin-biotin-peroxidase complex (Vector Elite ABC kit; Vector Laboratories) and 3,3’-diaminobenzidine substrate (Sigma) followed by counterstaining with haematoxylin (Dako) and examined by light microscopy. For immuno-fluorescence staining sections were incubated for 2 h with Cy3-conjugated (Jackson ImmunoResearch, West Grove, PA), AF488-, AF546- or AF633-conjugated (Invitrogen) secondary antibodies, counterstained either with DAPI (1 μg/ml; Sigma) or Sytox (250 nM; Molecular Probes), and mounted either in Mowiol or anti-fading (ProLong Gold; Molecular Probes) to be examined by fluorescence/confocal microscopes (Carl Zeiss and/or Leica Microsystems). As negative controls non-immunized serum from same species providing the primary antiserum were applied in adjacent specimen sections, using the same diluents and working dilutions, and following the same staining protocol, in order to check antibody specificity and unspecific background staining.

***Western blotting***

Total protein was obtained from lung and explants homogenized in lysing buffer (50 mM Tris-HCl pH 7.5, 150 mM NaCl, 1 mM EDTA, 1% Triton X-100, 0.1% SDS, 1 mM PMSF, 1μg/mL aprotinin, leupeptin and pepstatin, 1 mM NaF and Na_3_VO_4_). Proteins were separated by SDS-PAGE, transferred to PVDF membranes, blocked and incubated with primary antibodies listed and diluted in 2% BSA in TBST, as indicated in *Table S7*. β-Tubulin was used as loading control. For detection, secondary antibodies conjugated with HRP (Dako) were used and blots developed using ECL or ECL-Plus kits (Amersham). Films were scanned and images were analysed using a calibrated densitometer (Bio-Rad, Hemel Hempstead, Hertfordshire, UK). Quantitative results from Western blots were obtained after normalization of the densitometric data obtained from the specified samples of total or phosphorilated protein levels of a specific antibody, with densitometric values obtained for of β-Tubulin on the same blot.

**References**

1. Pichel JG, Fernandez-Moreno C, Vicario-Abejon C, Testillano PS, Patterson PH, et al. (2003) Developmental cooperation of leukemia inhibitory factor and insulin-like growth factor I in mice is tissue-specific and essential for lung maturation involving the transcription factors Sp3 and TTF-1. ***Mech Dev*** 120: 349-361.

2. Moreno-Barriuso N, Lopez-Malpartida AV, de Pablo F, Pichel JG (2006) Alterations in alveolar epithelium differentiation and vasculogenesis in lungs of LIF/IGF-I double deficient embryos. ***Dev Dyn*** 235: 2040-2050.
