# Supplementary material for: Transcriptome Analysis in Prenatal IGF1-Deficient Mice Identifies Molecular Pathways and Target Genes Involved in Distal Lung Differentiation
Source: PLoS One. 2013 Dec 31;8(12):e83028. doi: 10.1371/journal.pone.0083028 (PMC3877002; doi:10.1371/journal.pone.0083028)
Supplement: Supplementary file 10 [file pone.0083028.s003.doc]

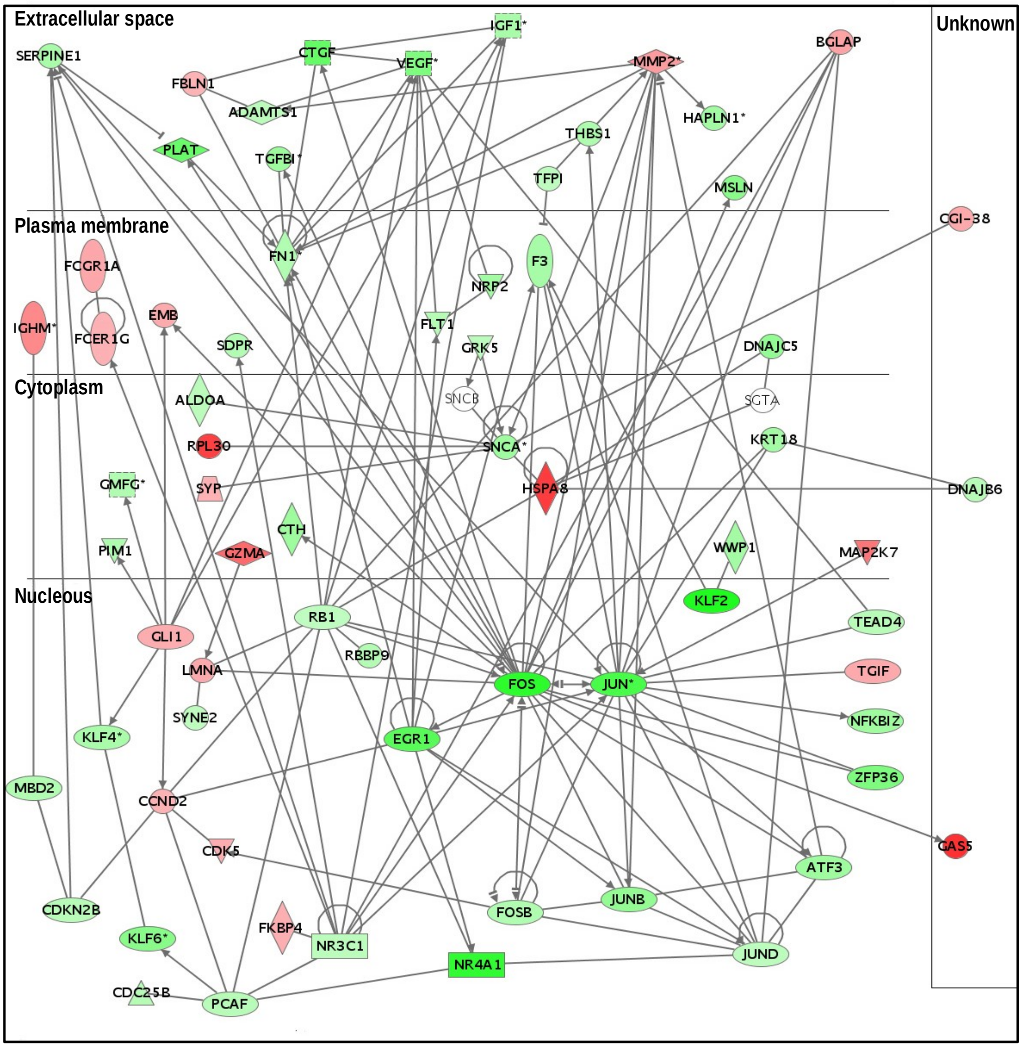
Extracellular space
Unknown
SERPINE1
CTGF
IGF1*
VEGF*
MMP2*
BGLAP
FBLN1
ADAMTS1
HAPLN1*
PLAT
TGFBI*
THBS1
TFPI
MSLN
Plasma membrane
CGI-38
FCGR1A
FN1*
F3
NRP2
IGHM*
FCER1G
EMB
FLT1
SDPR
GRK5
DNAJC5
Cytoplasm
ALDOA
SNCB
SGTA
RPL30
SNCA*
KRT18
GMFG*
SYP
HSPA8
DNAJB6
CTH
PIM1
GZMA
WWP1
MAP2K7
Nucleous
KLF2
RB1
GLI1
TEAD4
RBBP9
LMNA
FOS
JUN*
TGIF
SYNE2
EGR1
NFKBIZ
KLF4*
MBD2
ZFP36
CCND2
CDK5
GAS5
ATF3
CDKN2B
FKBP4
JUNB
FOSB
NR3C1
KLF6*
JUND
NR4A1
CDC25B
PCAF
