# Supplementary material for: Transcriptome Analysis in Prenatal IGF1-Deficient Mice Identifies Molecular Pathways and Target Genes Involved in Distal Lung Differentiation
Source: PLoS One. 2013 Dec 31;8(12):e83028. doi: 10.1371/journal.pone.0083028 (PMC3877002; doi:10.1371/journal.pone.0083028)
Supplement: Supplementary file 11 [file pone.0083028.s004.doc]

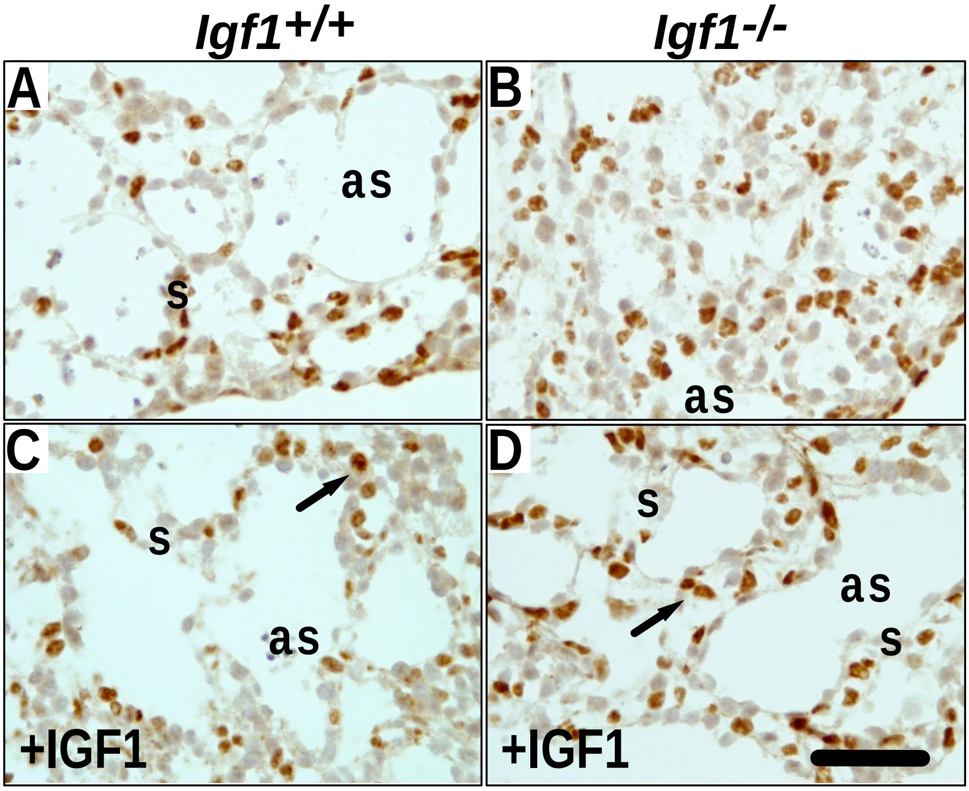
Igf1+/+
Igf1-/-
A
as
s
B
as
C
s
as
+IGF1
D
s
as
s
+IGF1
